# Expanding Chemical Space of Nucleic Acid Nanoparticles for Tunable Antiviral-Like Immunomodulatory Responses and Potent Adjuvant Activity

**DOI:** 10.1002/adfm.202515585

**Published:** 2026-01-07

**Authors:** Martin Panigaj, Hannah S. Newton, Jian Wang, Laxmi Pandey, Yelixza I. Avilla, Phong Nguyen, Morgan Chandler, Justin Halman, Yasmine Radwan, Stephanie Smith, Elijah Edmondson, Simone Difilippantonio, Chelsea Sanders, Bao Tran, Yongmei Zhao, Shaojun Xie, Edward Cedrone, Barry W. Neun, Meni Wanunu, Nikolay V. Dokholyan, Marina A. Dobrovolskaia, Kirill A. Afonin

**Affiliations:** 1 Chemistry and Nanoscale Science Program, Department of Chemistry, University of North Carolina At Charlotte, Charlotte, North Carolina, USA; 2 Nanotechnology Characterization Laboratory, Cancer Research Technology Program, Frederick National Laboratory For Cancer Research Sponsored By the National Cancer Institute, Frederick, Maryland, USA; 3 Department of Neurology, School of Medicine, University of Virginia, Charlottesville, Virginia, USA; 4 Department of Physics, Department of Bioengineering, Northeastern University, Boston, Massachusetts, USA; 5 MIMETAS US, Inc., Gaithersburg, Maryland, USA; 6 Molecular Histopathology Laboratory, Laboratory of Animal Sciences Program, Frederick National Laboratory For Cancer Research Sponsored By the National Cancer Institute, Frederick, Maryland, USA; 7 Animal Research Technical Support, Laboratory of Animal Sciences Program, Frederick National Laboratory For Cancer Research Sponsored By the National Cancer Institute, Frederick, Maryland, USA; 8 Frederick Sequencing and Genomics Core, Cancer Research Technology Program, Frederick National Laboratory For Cancer Research Sponsored By the National Cancer Institute, Frederick, Maryland, USA; 9 Frederick Sequencing and Genomics Core Bioinformatics, Bioinformatics Group, Bioinformatics and Computational Science, Frederick National Laboratory For Cancer Research Sponsored By the National Cancer Institute, Frederick, Maryland, USA

**Keywords:** cGAS, chemical modifications, cytokines, immunostimulation, NAMs, NANPs, RIG-I, TLRs, vaccine adjuvants

## Abstract

The rise of personalized medicine demands affordable biomaterials with tunable physicochemical properties, biological activities, and immune responses. We introduce a nucleic acid nanoparticle (NANP)-based platform designed to engage with the human immune system in a controllable manner. By varying the chemical composition while keeping the architectural parameters constant, we identified key factors that influence the immunorecognition and stability of NANPs. Chemical modifications provide additive functional regulation, while pairing NANPs with different delivery agents further enhances their immunomodulatory capabilities. Specific compositions boost T cell effector functions, promote B cell proliferation, and activate innate immune populations in human peripheral blood mononuclear cells. The resulting interferon responses and immune signatures mimic those triggered by viral infections. Notably, in vivo studies demonstrate that NANPs function as efficiently as traditional clinically used adjuvants, without increasing the risk of autoimmunity. This suggests that NANPs are a safe and effective tool for immunotherapy with promise for developing formulations targeting cancer and infectious diseases.

## 1 | Introduction

Pattern recognition receptors (PRRs), expressed in all mammalian cells, play a crucial role in the innate immune system by detecting pathogen-associated and danger-associated molecular patterns derived from pathogens and damaged or apoptotic cells, respectively [1]. The activation of PRRs leads to proinflammatory responses, which, depending on the triggering receptor, result in the elimination of the pathogen, tissue regeneration, and restoration of tissue homeostasis. This has made PRRs a promising target for therapies and vaccines, while also introducing translational challenges for certain types of biomolecular therapeutics.

As molecular architects of all life forms, RNA and DNA must be precisely differentiated between self and nonself via efficient sorting mechanisms that lead to PRR-mediated activation of protective immune responses when foreign nucleic acids are detected. This protective immunological sorting has profound implications for the development of all types of therapeutic nucleic acids (TNAs) [2, 3].

Over the last decade, TNAs emerged as innovative biomolecular agents that utilize a variety of cellular pathways to sense and target a wide range of diseases. However, despite the high clinical potential, confirmed by numerous FDA approvals [4–6, 7], the broader application of TNAs still faces significant challenges, including chemical instability, inefficient intracellular delivery, off-target effects, and immunotoxicity.

Programmability and modularity of nucleic acids allow for their controlled bottom-up self-assembly into the multi-stranded structures, called nucleic acid nanoparticles, or NANPs [8] that can be rationally designed to advance the capabilities of TNAs. NANPs show potential for tighter control over stability, biodistribution, and synchronized delivery of precisely organized TNA cocktails, while ensuring their conditional activation within diseased cells [9–11].

To advance NANP technologies, understanding their largely unknown immunological profiles is essential for clinical translation. We conducted the first systematic studies of NANP immunorecognition, revealing that the immunostimulatory activity depends on the size, shape, and composition of NANPs [12–14, 15]. Our findings suggested that NANPs can serve not only as delivery scaffolds for multiple TNAs but also as standalone therapeutics for next-generation immunotherapies with predictable immunorecognition [16]. While the individual monomers comprising NANPs have intrinsic immunostimulatory properties of RNA and DNA, their assembly into macromolecular architectures introduces structural complexity and dimensionality that enables tighter control over immunostimulation both quantitatively (e.g., by modulating the magnitude of the innate immune response) and qualitatively (e.g., by shaping the spectrum of innate immune mediators) [13].

Previously, detailed experimental studies have identified three-dimensional architectures composed of RNA to be the most immunostimulatory. Among them, six-stranded RNA cubes, delivered into immune cells, were the most potent activators of type I and type III interferons (IFN) [13]. We further demonstrated that the spectrum of proinflammatory mediators can be controlled by the type of carrier used to deliver NANPs into cells [17–19].

In the present study, we investigated how chemical modifications of oligonucleotide components affect the physicochemical, mechanical, and immunostimulatory properties of NANPs. We conducted extensive immunophenotyping and mechanistic studies to identify the PRRs and molecular pathways involved in NANP-mediated immunostimulation and carried out computational studies to further elucidate NANP-PRR interactions. We then assessed the impact of NANPs on epithelial barrier function using new approach methodologies (NAMs) such as innovative organoid 3D cultures. Finally, we evaluated NANPs’ adjuvant properties in vivo and gained insight into their immunological safety by examining the potential for autoimmunity induction, an adverse effect limiting the clinical use of traditional low-molecular-weight TLR agonists to topical applications.

## 2 | Materials and Methods

### 2.1 | Reagents

RPMI-1640 medium and its supplements, Fetal Bovine Serum (FBS), Penicillin Streptomycin Solution, L-glutamine, along with Ficoll-Paque Premium and Phosphate Buffered Saline (PBS) were purchased from Cytiva Life Sciences (Marlborough, MA, USA). Hank’s Balanced Salt Solution (HBSS) was obtained from Gibco (Gaithersburg, MD, USA). ViaStain Acridine Orange (AO)/Propidium Iodide (PI) staining solution was obtained from nexcelom bioscience (Lawrence, MA, USA). Phytohemagglutinin (PHA-M) and Phorbol 12-myristate 13-acetate (PMA) were obtained from Sigma-Aldrich (Burlington, MA, USA). Ionomycin was obtained from STEMCELL Technologies (Vancouver, CA). Oligodeoxyribonucleotide, human TLR9 Ligand (ODN2216), and Lipopolysaccharide From *E. coli* K12 (LPS) were obtained from InvivoGen (San Diego, CA, USA). Additional reagents were needed for the NANP Treatment of PBMC. Lipofectamine MessengerMax (LMM) was obtained from Invitrogen (Waltham, MA, USA). Opti-MEM Reduced Serum Medium Was Obtained From Gibco (Gaithersburg, MD, USA). The staining process of the samples and flow cytometry required some reagents additional to the ones already listed above. EBioscience flow cytometry staining buffer and UltraComp eBeads Plus compensation beads were obtained from Invitrogen (Waltham, MA, USA). Antihuman mouse antibodies used for flow cytometry (labeling antibodies and isotype control antibodies) were obtained from BioLegend (San Diego, CA, USA) or Invitrogen (Waltham, MA, USA) and are listed in NCL Protocol ITA-37.1 and ITA-37.2 [20, 21]. Paraformaldehyde (PFA) 20% solution was purchased from Electron Microscopy Science (Hatfield, PA, USA). The flow cytometer solutions NovoFlow, NovoRinse, and NovoClean were all obtained from Agilent Technologies (Santa Clara, CA, USA)

### 2.2 | Synthesis of NANPs’ Monomers

Cube monomers were synthesized in the laboratory by in vitro run-off transcription (RNA, 2’F, 2’OMe, and *ψ*). While RNA (5’OH) and all DNA (including phosphorothioate DNAs) monomers were either purchased from Integrated DNA Technologies (IDT) or ThermoFisher. Run-off transcription was performed on PCR-amplified DNA. For transcription, two different protocols were used for either standard RNA or 2’ ribose-modified RNA. For Standard RNA, a solution of 80 mM HEPES-KOH (pH 7.5), 2.5 mM spermidine, 50 mM DTT, 2 mM MgCl_2_, 25 mM rNTPs, 0.2 μM DNA templates, and ~100 Units/μL of T7 RNA polymerase (purified in-house) was Incubated for 4–16 hrs at 37°C. For 2’ ribose modified RNAs, transcription was performed in a solution of 40 mM Tris-HCl (pH 8.0), 30 mM MgCl_2_, 6 mM spermidine, 6 mM 2’ ribose modified NTPs, 10 mM DTT, and RGVG-WT-T7 RNA polymerase (mutant RNA polymerase capable of incorporating 2’ ribose modified NTPS)

### 2.3 | Assembly of NANPs

All NANPs were produced by mixing six cognate strands at an equimolar ratio in one-pot assembly. Initial thermal denaturation for 2 min at 95°C was followed by incubation for 2 min at 45°C, then assembly buffer was added to reach a final concentration of 89 mM Tris-borate, 2 mM MgCl_2_, and 50 mM KCl. The samples were then incubated for an additional 30 min at 45°C. Samples were then stored at 4°C indefinitely. To confirm assemblies, samples were assessed with Native-PAGE, 8% 37.5:1 Acrylamide/Bis-acrylamide, with total ethidium bromide staining. Throughout the manuscript, unmodified NANPs are referred to as RNA cubes (assembled from in vitro transcribed strands) and DNA cubes, whereas NANPs containing chemical modifications are designated by the type of modification (e.g., 2’F, 2’OMe, *ψ*)

### 2.4 | Atomic Force Microscopy

5 μL (50 nM) of each NANP was deposited on APS-modified mica, incubated for ~2 min, and airdried, as described previously according to established protocols [22]. Briefly, AFM was performed using a MultiMode AFM Nanoscope IV system (Bruker Instruments, Santa Barbara, CA) in tapping mode. The images were recorded with a 1.5 Hz scanning rate using a TESPA-300 probe from Bruker with a resonance frequency of 320 kHz and spring constant of about 40 N/M. Images were processed by the FemtoScan online software package (Advanced Technologies Center, Moscow, Russia)

### 2.5 | Thermodynamic Stability of NANPs

Temperature gradient gel electrophoresis (TGGE) was conducted at 6% polyacrylamide gels (37.5:1) for 5 min (200 V) prerun before being placed on the TGGE instrument. The gels were then run for 20 min at 200 V with the range of temperatures indicated on the gels. Ethidium bromide total staining was used for visualization and assessment of melting temperatures.

### 2.6 | Nuclease Stability of NANPs

Relative stability from nuclease degradation was compared by incubating each NANP (500 nM) in 10% fetal bovine serum (FBS) and measuring the intactness of the samples over the course of 1 h or 5 min at 37°C. At specific time points, aliquots were removed, mixed thoroughly with native-PAGE loading buffer (89 mM trisborate, 2 mM MgCl2, 50 mM KCl, 30% glycerol), and snap frozen on dry ice. Once all samples were collected, each sample was rapidly thawed and loaded into a gel (8% 37.5:1 acrylamide/bisacrylamide). Samples were assessed using ethidium bromide total staining or SYBR Green RNA II.

### 2.7 | Nanopore Characterization of NANPs

Nanopores were fabricated in freestanding, high-stress silicon nitride membranes (20 × 20 μm) at the center of each 5 × 5 mm^2^ Si chip using JEOL 2010F transmission electron microscope as described previously [23]. Chips were mounted onto the custom-designed flow cell with quick-curing silicone elastomer (Ecoflex 00–35 Fast) after being cleaned with hot piranha, followed by rinsing with water and drying with nitrogen gas. Each chamber of the flow cell, separated by the chip, was filled with electrolyte and equipped with an Ag/AgCl electrode. NANP solution was added to the *cis* (grounded) chamber, and positive voltage was applied to the *trans* chamber. Ionic current signals were measured using a Chimera VC100 amplifier (Chimera Instruments LLC) [24] sampled at 4.17 MHz. Pyth-Ion software (https://github.com/rhenley/Pyth-Ion/) was used for data processing, and data were digitally low-pass filtered at 250 kHz.

### 2.8 | Mechanistic Study Using PRR Reporter Cell Lines

Reporter cells for several innate immune receptors and signaling pathways were used to assess NANP immunostimulation after activation of PRRs. The cell lines HEK-Blue hTLR 3, HEK-Blue hTLR 7, HEK-Blue hTLR 9, HEK-Lucia RIG-I, and THP1-Dual were purchased from InvivoGen. For all cells, the media was prepared as recommended by the manufacturer. For each cell line, 40 × 10^3^ cells/well were plated in a 96 well plate and allowed to adhere overnight. The suspension THP1-Dual cells were either transfected immediately after plating or 24 h later. For transfections, 0.5 μL of Lipofectamine 2000 (L2K) per well was mixed with each NANP and incubated for 30 min at room temperature. Following incubation, samples were diluted into fresh media to reach a final concentration of 5 nM. The media on the cells was aspirated, and the media containing the NANP/carrier complex was then used to replace it. The cells were then incubated for 24 h at 37°C, 5% CO_2_. For HEK-Blue and THP1-Dual (NF-*κ*B pathway) cells, 20 μL of cell supernatant was mixed with 180 μL of Quanti-Blue and incubated at 37°C for ~70 min, then the absorbance was measured at 620 nm. For HEK-Lucia and THP1-Dual (IRF pathway) cells, 20 μL of cell supernatant was mixed with 50 μL of Quanti-Luc, and the luminescence was read immediately.

### 2.9 | Research Donor Blood

Fresh human blood was obtained from healthy donor volunteers under the IRB-approved NCI-Frederick protocol OH99CN046D. This protocol is designed to establish and operate a Research Donor Program that meets the requirements for the protection of human subjects from research risks, as detailed in the NIH Multiple Projects Assurance with the OPRR, and is in compliance with the Code of Federal Regulations, Public Welfare, 45 CFR 46: Protection of Human Subjects. Office for Protection from Research Risks, U.S. Government Printing Office, Washington, DC, 1997. Informed consents were obtained from all human participants of the NCI-Frederick Research Donor Program. The blood was collected in vacutainers containing lithium heparin as an anticoagulant (Becton Dickinson, Franklin Lakes, NJ, USA) and used within 1–2 h after collecting for the isolation of peripheral blood mononuclear cells (PBMCs) for immunophenotyping, cytokine analysis, and single-cell sequencing studies.

### 2.10 | PBMCs Isolation and Treatment with NANPs

#### 2.10.1 | PBMC Isolation

Fresh anticoagulated whole blood was diluted with an equal volume of PBS and then layered over Ficoll-Paque Premium solution. Density gradient centrifugation was then used to separate out the mononuclear cell layer. This mononuclear cell layer was collected and washed twice with HBSS. The PBMCs were resuspended in RPMI-1640 medium supplemented with 10% heat-inactivated FBS, 100 U/mL penicillin, 100 μg/mL streptomycin, and 2 mM L-glutamine (complete RPMI-1640 medium), counted, and the cell concentration was adjusted to 1.25 × 10ˆ6 cells/mL. The PBMCs isolation procedure is detailed in the NCL protocols ITA-10 and ITA-37.2, and also described in our previous studies [25].

NANPs Complexation with Carriers Prior to PBMCs Incubation for Immunophenotyping, Cytokines and Single-Cell Sequencing Analysis. PBMCs were cultured at a final concentration of 1 × 10ˆ6 cells/mL (final volume, 1 mL) in a 24-well plate in a 37°C/95% CO_2_ incubator for approximately 20 h. Untreated (or unstimulated) PBMC were used as negative controls and consisted of 800 μL of PBMCs (1.25 × 10^6^ cells/mL) and 200 μL of complete RPMI-1640 media. Positive controls consisted of PBMCs activated with 20 ng/mL LPS + 10 μg/mL PHA-M, 50 ng/mL PMA + 1 μg/mL Ionomycin, or 5 μg/mL ODN2216 + 10 μg/mL PHA-M. Stock NANPs were complexed with the lipofectamine carrier (LMM for immunophenotyping, and LMM or L2K for cytokine analysis). An aliquot of 100 μL of 1 μM NANP stock was combined with 20 μL of the carrier and mixed well by pipetting. Each NANP/carrier complex was then incubated for 5–30 min at room temperature, followed by dilution with 1.88 mL of OptiMEM medium (total volume of 2 mL, at a concentration of 50 nM NANP). The carrier-only control was prepared in the same manner: OptiMEM medium was combined with LMM (at a ratio of 5 parts medium to 1 part LMM) and then incubated at room temperature for 5–30 min. It was then diluted further with OptiMEM medium to obtain LMM at the same concentration as in the complexed samples. The complexed NANPs or prepared LMM control (200 μL) were then added to the PBMC (800 μL, 1.25 × 10ˆ6 cells/mL) at a dilution of 5 (MRD5). The final concentration of the NANPs in each PBMC sample was 10 nM in 1 mL. After 24 h of incubation, the supernatants were collected and stored at a nominal temperature of −20°C for subsequent analysis of cytokines. The cells were then used for immunophenotyping or single-cell sequencing, as detailed below.

### 2.11 | Cytokine Analysis

Cytokines in PBMC culture supernatants were detected using custom multiplex ELISA kits (Quansys Biosciences, Logan, UT, USA), as detailed in the NCL protocol ITA-27 [26]. Preparation of culture supernatants is detailed in the NCL protocol ITA-10 and was described by us earlier [13, 24].

### 2.12 | Immunophenotyping

The immunophenotyping protocol is detailed in NCL protocol ITA-37.2 [20, 21, 26, 27]. Briefly, untreated (negative control), activated (positive controls), and NANP-treated PBMCs were stained with antibodies specific to individual cell populations and their activation markers. Master mixes for each panel were prepared using the optimal antibody dilution corrected for cell concentration and staining volume for each individual antibody. Each panel contained a labeling antibody master mix and an isotype control master mix (both at equal concentrations). The cell samples were washed twice with 1X PBS, and all appropriate samples (samples to be stained with labeling antibody master mixes) were stained with Zombie Aqua dye for 30 min at room temperature. The samples were then washed twice with staining buffer and incubated for 30 min with the appropriate amount of corresponding master mix or staining buffer (final staining volume: 100 μL). Finally, the samples were washed with staining buffer, fixed with 2% PFA for 15 min at room temperature, washed twice more with staining buffer, resuspended in 500 μL staining buffer, and stored at 4°C until data acquisition. The data acquisition was performed on the NovoCyte 3005 (Agilent Technologies, Santa Clara, CA, USA). Detailed descriptions of the instrument calibration and gating strategies are available in the supplementary materials. FCS Express 7, Microsoft Excel, and GraphPad Prism were used for data analysis and visualization. This experiment employed PBMCs from three healthy donors as follows: N6T3, P1F3, and O8E8 blood was used for analysis of all treatments against the negative (untreated cells) control; due to the unavailability of the donor O8E8, additional analysis of untreated cells vs. LMM carrier was done using blood of donors N6T3, P1F3, and G2S7.

### 2.13 | Single-Cell Sequencing

PBMCs of one donor (P1F3) selected as the most reactive to NANPs in the immunophenotyping experiment were cultured with LMM-complexed RNA cubes for 24 h. The isolation of single cells, purification, library preparation, and sequencing were performed at the NCI-Frederick Sequencing and Genomics Core. The single-cell RNA-seq libraries were prepared using a Chromium Next GEM Single Cell 5’ reagent kit v2 dual index protocol according to the manufacturer’s instructions. The libraries were pooled and sequenced on a NextSeq2000 instrument with P2 flowcell (Illumina) using 200 cycles for dual indices, 26 cycles for Read1, and 90 cycles for Read2 according to the manufacturer’s instructions.

### 2.14 | Bioinformatics Analysis of Single-Cell Sequencing Data

Single-cell data pre-processing and gene and transcript counting were performed using 10x Genomics Cellranger v7.1.0 software. The pre-built reference (refdata-gex-GRCh38–2020-A) was downloaded from 10x website via the URL: https://www.10xgenomics.com/support/software/cell-ranger/downloads#reference-downloads. Seurat software package (v4.3.0) [28, 29] was used for further pre-processing, read normalization, scaling data, clustering, marker gene identification, visualization, and differential analysis between clusters or sample conditions. Cell type annotations were performed using SingleR (v2.0.0) [30] with a reference constructed from Human Primary Atlas (HPCA) data and Blueprint and ENCODE data. The Gene Set Enrichment Analysis was performed using the fgsea (v1.28.0) [31] R package. EnhancedVolcano (v1.16.0) [32] was used to generate volcano plots.

### 2.15 | OrganoPlate Culture

To assess the uptake of modified cubes in 3D culture, 3L64 OrganoReady Colon Caco-2 (Mimetas BV, The Netherlands) plates were prepared and used. First, collagen I is seeded, then Caco-2 cells were seeded to form tubules against collagen I [33–36]. The Caco-2 cultures form leak-tight tubules used to assess the barrier integrity of the 3D culture [37].

Alexa 488 labelled NANPs were assembled and characterized, as described above. This experiment assessed the uptake of 7 different modified cubes and their corresponding unmodified RNA or DNA cubes. The cubes were complexed with Lipofectamine 2000 Transfection Reagent (L2K) (Thermo Fisher Scientific, USA) and DOTAP/DOPE, and incubated at RT for 30 min. Prior to transfection, the final volume was reached by adding Caco-2 media. The final concentration of cubes tested was 100 nM. Controls of this experiment included vehicle carriers only (L2K, DOTAP/DOPE), and Al488 labelled monomer of RNA and DNA cubes complexed with each carrier. Untreated cells were used as a negative control. The experiment was done in triplicate. Before transfection, the chips’ inlets and outlets were aspirated, 50 μL of treatment was added to each right inlet and outlet, and 50 μL of fresh Caco-2 media was added to the left inlets and outlets. The OrganoPlate was incubated on the OrganoFlow (Mimetas BV, The Netherlands) at 14°/8 min settings in a 37°C, 5% CO2 incubator for 72 h.

### 2.16 | Transepithelial Electrical Resistance (TEER)

To measure the tightness of the Caco-2 tubules, TEER values were measured before transfection, and every 24 h after transfection up to 72 h. TEER values indicate the toxicity of modified and unmodified RNA and DNA cubes to the cells by assessing their induced disruption to the barrier over time. TEER values were measured via OrganoTeer (Ω*cm2) (Mimetas BV, The Netherlands) after the OrganoPlate was equilibrated to RT for 30 min. TEER was measured before transfection, and every 24 h after transfection up to 72 h, and the values were recorded for analysis.

### 2.17 | Staining and Imaging

The 72 h after transfection, at the endpoint of the experiment, the cells were washed and fixed using 3.7% formaldehyde (Sigma) in HBSS with Calcium and Magnesium (Gibco) for 15 min. The cells were then washed twice for 5 min with PBS (Gibco), then stained immediately. For nucleus staining, NucBlue Fixed Cell ReadyProbes Reagent (Thermo Fisher, USA) was used following the manufacturer’s protocol and incubated on a RT rocker for 15 min. After staining, the stained wells were aspirated and washed with PBS for 1 min. Finally, all wells were aspirated, and 50 μL of PBS was added to all wells, including the observation windows and gel inlets. Cytation 5 Multi-Mode Microplate (BioTek, USA) was used for imaging the OrganoPlate, GFP, and DAPI filters were used at 4X. Image analysis was done with ImageJ. Uptake data at 72 h were quantified using ImageJ.

### 2.18 | In Vivo Autoimmunity Assessment

The study was conducted according to the NCI-Frederick ACUC-approved animal study protocol in 8-week-old SJL/J females as described previously [38]. NCI-Frederick is accredited by the Association for Assessment and Accreditation of Laboratory Animal Care (AAALAC) International and follows the Public Health Service Policy for the Care and Use of Laboratory Animals. Animal care was provided in accordance with the procedures outlined in the “Guide for Care and Use of Laboratory Animals” (National Research Council, 1996; National Academy Press, Washington, D.C.). The animals were administered a single dose of either negative control (PBS), positive control (pristane), DNA cubes, RNA cubes, or a combination of DNA cubes and PC or RNA cubes and PC, in a volume of 0.5 mL per animal. The NANPs concentration of the injected solution was 10 nM. Pristane was used as a commercially provided stock without further dilution (cat. #P1403, Sigma-Aldrich, USA). The blood was collected on weeks 8, 16, and 20 postinjection and analyzed for the presence of anti-dsDNA antibodies using commercial ELISA kits (Chondrex, cat# 3031 and 3032). At the end of the study, the animals were euthanized; the kidney tissues were fixed in 10% NBF and paraffin sections stained with standard H&E and periodic acid-Schiff (PAS). Slides were read and interpreted by a board-certified veterinary pathologist (EFE). H&E and PAS-stained renal sections were evaluated, and longitudinal sections from both kidneys were examined. Grading was based on the most severe kidney section. Grading was performed for glomerular changes, inflammatory infiltrates, and tubular changes as follows: 0-normal; 1-minimal; 2-mild; 3-moderate; 4-marked. Inflammatory infiltrates are graded as 0, within normal limits; 1, minimally increased inflammatory infiltrates composed predominantly of lymphocytes and plasma cells often focally forming 3–5 cell thick perivascular cuffs; 2, mildly increased inflammatory infiltrates that are multifocal; 3, moderately increased inflammatory infiltrates that form thick perivascular cuffs multifocally that are prominent with the 4x objective; 4, markedly increased inflammatory infiltrates that widely separate vessels from adjacent renal parenchyma. Tubular changes are graded as follows: 0, within normal limits; 1, minimal tubular degeneration, often focal; 2, mild tubular degeneration present with multifocal regions containing tubular degeneration and regeneration and focal necrosis; 3, moderate tubular changes contain tubular degeneration and multifocal necrosis; 4, marked tubular changes including tubular necrosis multifocally. The mesangial expansion was evaluated and graded into four categories: 0, no mesangial expansion; 1, minimal changes; 2, mild mesangial expansion (mesangial matrix wide < 2 nucleus diameter); 3, moderate mesangial expansion often with crescentic glomeruli (mesangial matrix wide < 4 nucleus diameter), and 4, severe mesangial expansion (> 4 nucleus diameter). Shown on the graph is a cumulative score of all changes.

### 2.19 | NANPs Adjuvant Activity In Vivo

The study was carried out according to the UNC Charlotte IACUC-approved animal study protocol. We investigated the ability of RNA cubes complexed with DOTAP/DOPE to function as an experimental adjuvant, compared to the standard adjuvant Alum (Alhydrogel adjuvant 2%, Invivogen). As a model antigen, we used ovalbumin (OVA; EndoFit Ovalbumin, InvivoGen). The OVA was administered through rare footpad injection either alone or in the presence of Alum adjuvant or RNA cubes in a total volume of 30 μL [39]. The blood was collected on the day of administration and then 7 and 21 days after the immunization. Isolated serum was stored at −20°C to later monitor the titer of total OVA-specific antibodies by ELISA (Mouse Anti-Ovalbumin Ig’s (total (A+G+M) ELISA Kit, Alpha Diagnostic).

### 2.20 | Building the Initial Structures of DNA, RNA Cubes, and Cube/Protein Complexes In Silico

We previously constructed the 3D structures of both DNA and RNA cubes. To generate the RNA cube with a 5’ triphosphate (5’PPP), we used the 3D structure of ATP as a template. We aligned the 3D coordinates of atoms in the sugar ring of ATP to the ring of 5’ residue of each RNA chain. The aligned atoms included O5,’ C5,’ C4,’ O4’, and C3.’ After aligning, we transferred the following atoms with their 3D coordinates from ATP to the 5’ end of the RNA cube: PG, O1G, O3G, O2G, PB, O1B, O2B, O3B, PA, O1A, O2A, and O3A. For the RNA cube with pseudouridine, we modified the uridine (U) residues by renaming the following atoms: N1 to C5, O4 to O2, O2 to O4, C4 to C2, C2 to C4, and C5 to N1. To create the RNA cube with 2’-O-methyl (2’OMe) modifications in all uridines and cytidines (Us and Cs), we found a 2’OMe-modified RNA nucleotide template from the RCSB PDB. We aligned the sugar ring of this template to each U and C and then copied the 2’OMe group accordingly. The aligned atoms were O3,’ C3,’ C4,’ C2,’ C1,’ and O2.

For the RNA cube with 2’-fluoro (2’F) modifications in all of Us and Cs, we renamed the O2’ atom to fluorine (2’F). To build the DNA cube with phosphorothioated thymidine (PS T), we replaced the OP2 or O2P atoms in deoxythymidine (DT) with sulfur (S). For the DNA cube with uridines (Us) at the corners, we replaced the DT residues at positions 7, 8, 9, 20, 21, 22, 33, 34, 35, 46, 47, and 48 in each chain with Us by removing the C7 atom.

### 2.21 | Modeling NANP-PRR Complexes

To model the NANP-PRR complexes, we used the AlphaFold3 web server to generate the initial structures of the TLR7/RNA cube and RIG-I/RNA cube complexes. We then modified the RNA cubes as described above. Since AlphaFold3 could not model the docking between the cube and TLR7, we manually docked various RNA cube fragments to TLR7. These fragments included: 5’-UUUCACG, 5’-GGCAACUUU, 5’-UUUGUUGCCCGUGUUU, 5’-GGCAACUUUGAUCCC, 5’-UCGGUUUAGCGCC, 5’-GGCCUUUUCUCCC, and 5’-ACACUUUCACG. AlphaFold3 was able to successfully dock the RNA cube to RIG-I.

### 2.22 | Docking and MD Simulations

Docking was performed with the MedusaDock software [40–42]. Parameters were initialized with the default values. MD simulations were conducted using the Amber 18 software suite [43]. The initial complexes were positioned within periodic boundary conditions and solvated explicitly using the TIP3P water model. To achieve equilibrium, the systems were stabilized at 310 K and 1 bar under isobaric conditions, applying the Parrinello-Rahman barostat with a coupling constant of 0.1 ps. The equilibration phase lasted for 10 ns with a time step of 2 fs. Subsequently, production simulations were performed for 200 ns with the same time step, while all covalent bonds were constrained using the LINCS algorithm. Long-range electrostatic interactions were calculated with a cutoff distance of 12 Å. The Amber14SB force field was employed throughout the simulations, and visualizations of the structures were rendered using PyMOL (Schrodinger, L. L. C. The PyMOL molecular graphics system. Version 1, 0–0 (2010).

### 2.23 | Statistical Analysis

Experimental data were normalized to untreated controls and are presented as mean ± SEM (or SD). The number of biological replicates is specified in the corresponding figure legends. Statistical analyses were performed using GraphPad Prism, with *p* < 0.05 considered statistically significant.

## 3 | Results and Discussion

### 3.1 | Selection of Chemical Modifications and Delivery Carriers

Chemical modifications of viral RNA can modulate the activity of PRRs in favor of the virus, enhancing its survival by influencing RNA structure, stability, and other RNA-associated pathways [44–47]. Incorporating natural or artificial modifications, including backbone alterations, into synthetic nucleic acids preserves their ability to self-assemble while improving stability, enhancing binding interactions, and enabling site-specific attachment chemistry. Many of the substituents used in nucleoside structures are also naturally present in eukaryotic cells, where they participate in diverse biological pathways, suggesting the inherent biocompatibility of such modifications [48, 49]. Consequently, most TNA formulations that advance to clinical trials include multiple chemical modifications [2, 47, 50].

**FIGURE 1 |:**
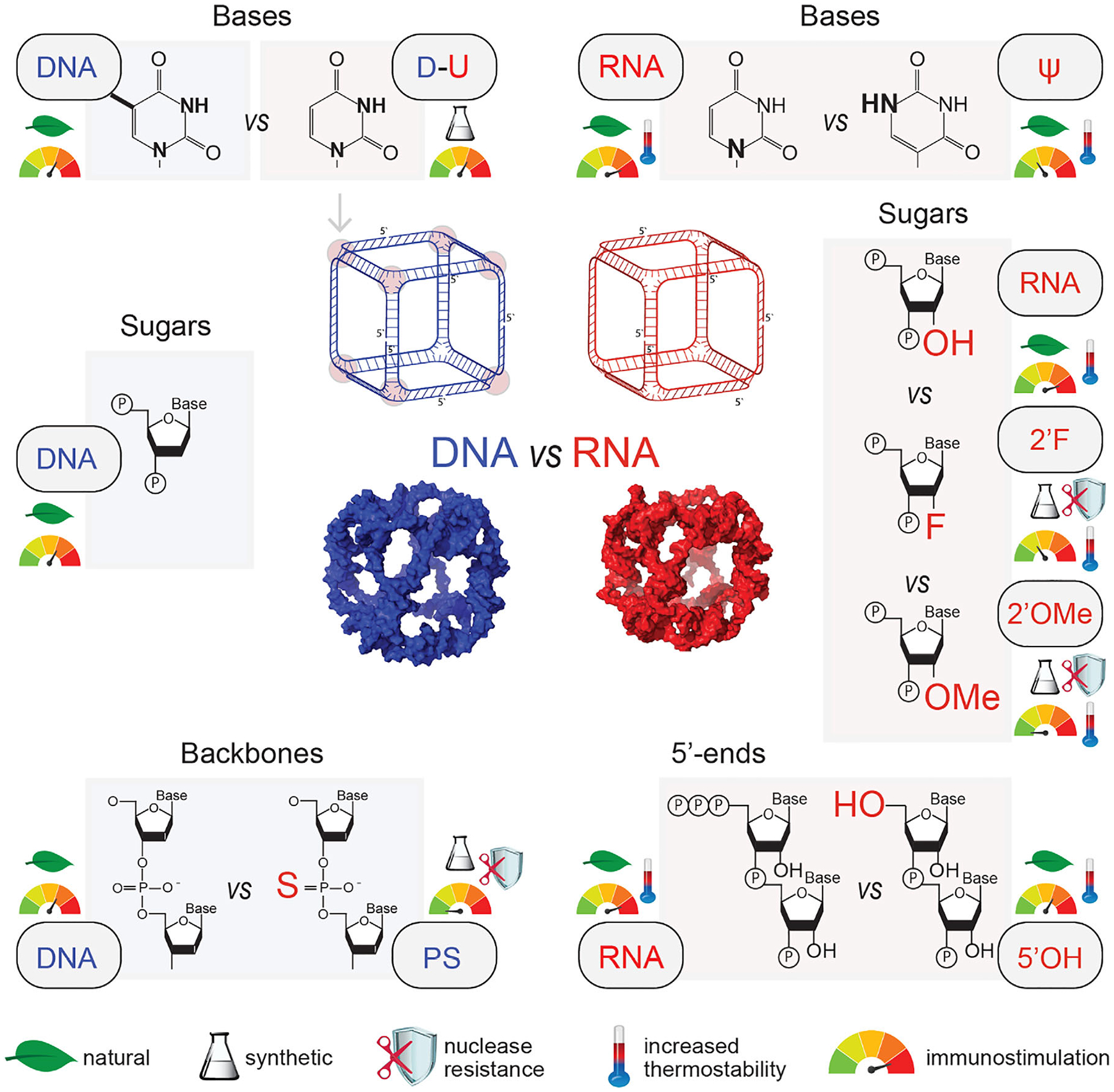
The spectrum of modifications, their occurrence, relative nuclease resistance, thermal stabilities, and immunostimulation. In total, nine different compositions of NANPs were tested: DNA cubes (DNA), DNA cubes with backbone phosphorothioate modifications (PS), and DNA cubes containing ssUs in the corners (D-U). RNA cubes with hydroxyl group at 5’ end (5’OH), RNA cubes with triphosphate group on 5’ end (RNA). The rest of RNA cubes had 5’-triphosphates and chemical modifications at ribose, where 2’-hydroxyl was substituted with either fluorine (2’F) or 2’-O-methyl (2’OMe) groups or uridine is substituted with pseudouridine (*ψ*).

To investigate how chemical modifications of the identical sequences of oligonucleotide components of NANPs influence their physicochemical and immunostimulatory properties, we constructed a panel of NANPs with the following spectrum of representative chemical compositions and modifications: (i) RNA vs. DNA, (ii) DNA with phosphodiester (DNA) vs. phosphorothioate backbone (PS); (iii) DNA with Ts (DNA) vs. DNA with ssUs in the NANPs’ corners (D-U); (iv) RNA with Us (RNA) vs. RNA with pseudo-Us (*ψ*); (v) unmodified RNA (RNA) vs. 2’F- *vs*. 2’OMe-modified cubes; (vi) RNA containing 5’ triphosphates (RNA) vs. RNA with 5’OH groups (5’OH) (Figure 1 and Figure S1).

The spectrum of modifications was selected to encompass the potential interaction with a broad range of PRRs that sense nucleic acids in endosomes and the cytosol. Furthermore, the range of selected modifications covers the backbone, sugar, and base compositions of nucleotides and represents both naturally occurring and synthetic analogs used in biomedicine. RNA without 5’-triphosphates (5’PPP), containing only 5’OH, doesn’t trigger the cytosolic RIG-I sensor, while RNA with 5’PPP is a potentially strong activator of RIG-I [51]. RNA with 5’OH termini is prepared synthetically in vitro, and in cells, this type of modification occurs as a product of endonuclease cleavage [52]. The 5’PPP RNA is produced by some viruses, but cells can produce this type of RNA, too [53, 54]. DNA with a phosphodiester bond is recognized by Toll-like receptor 9 (TLR9), a primary receptor for DNA in endosomes, while DNA in the cytoplasm is recognized by cyclic GMP-AMP synthase (cGAS) that triggers the stimulator of interferon genes (STING) pathway [55, 56]. DNA with phosphorothioate backbone (PS) modification enhances the resistance of DNA against degradation by nucleases, increasing its half-life. Phosphorothioate (PS) modifications are widely employed in the production of antisense oligonucleotides (ASOs) to enhance their binding affinity to target RNAs. These modifications also increase ASOs’ interactions with proteins, which can significantly influence their distribution across tissues, cellular uptake, and intracellular trafficking [57]. RNA modified with pseudouridine (*ψ*) is the most abundant post-transcriptional RNA modification [58]. In therapeutic mRNAs, *ψ* enhances mRNA stability and decreases immunogenicity [59]. Synthetic 2’F RNA modification has found numerous beneficial applications in the production of ribozymes, ASOs, siRNAs, etc [60]. 2’OMe is a common natural modification in RNA with high impact on many cellular processes, such as translation, epigenetic gene regulation, and self-versus nonself-recognition [61]. The 2’OMe modification plays together with 2’F, PS, and *ψ* crucial role in development and application of therapeutic nucleic acids, and many approved therapeutics contain one or combination of more types of modifications.

Since carriers are required for intracellular delivery of NANPs and their exposure to different PRRs, we delivered chemically modified NANPs by commonly used lipofectamine and DOTAP/DOPE agents [62, 63]. DOTAP and DOPE are components in lipid-based formulations for non-viral gene/oligonucleotide delivery, and their combination is frequently adopted in pre- and clinical studies for mRNA vaccines [64–66].

### 3.2 | Thermodynamic and Chemical Stability of NANPs Depends on Their Composition

**FIGURE 2 |:**
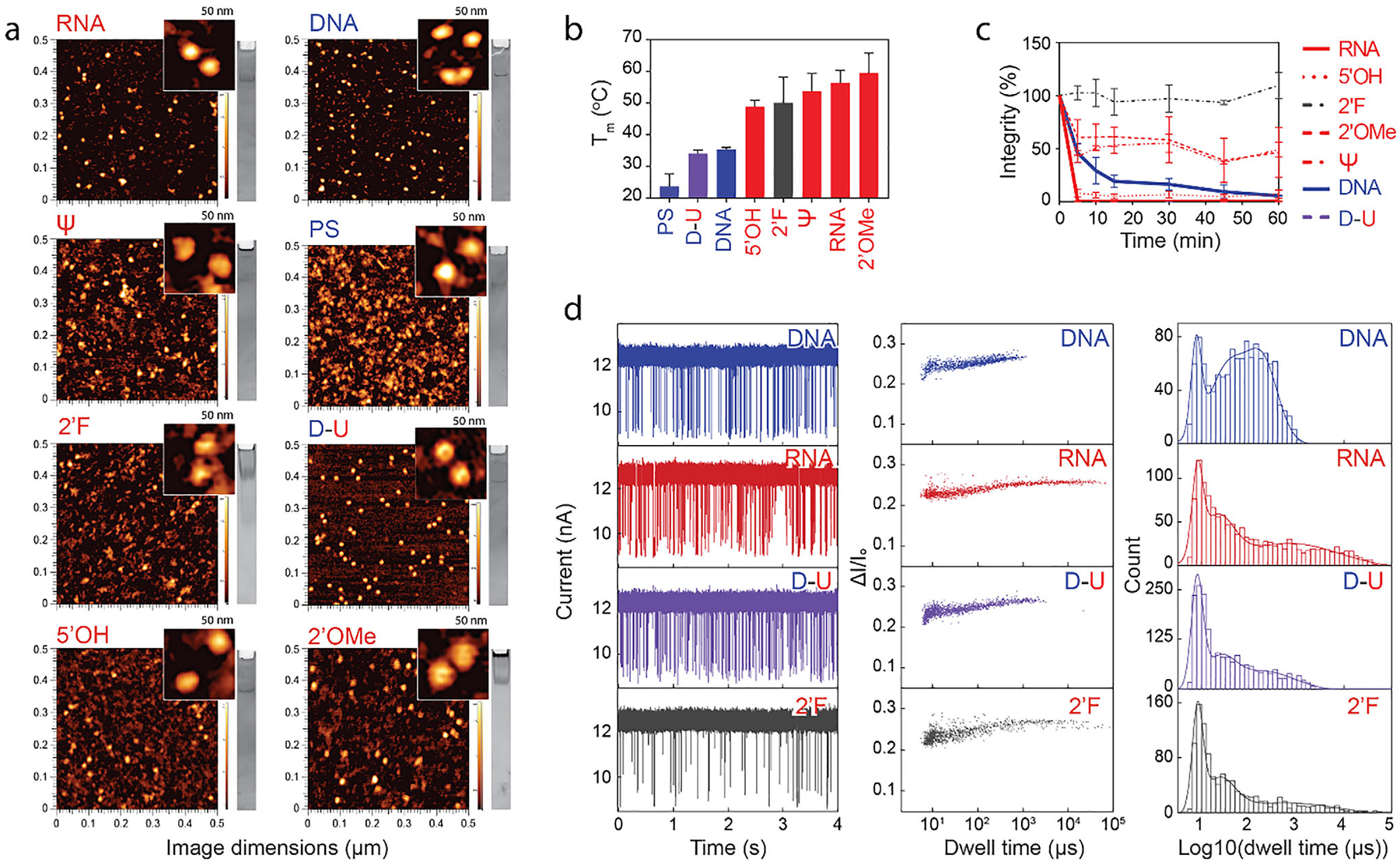
Physicochemical characterization of NANPs. (a) Representative AFM and native-PAGE results confirming NANP assemblies. (b) Thermal stability assessed by TGGE and (c) nuclease resistance evaluated by incubating NANPs in 10% fetal bovine serum (FBS) at 37°C for 60 min. (d) Characterization of different NANPs with a solid-state nanopore with current traces at 1 V (left), scatter plot of fractional current blockade vs. dwell time (middle), and histograms of log10 (dwell time) (right) are shown. The cubes were analyzed using a pore with ~10.2 nm diameter (TEM-based). The concentration of DNA and RNA cubes was 4.2 nM, while that of the D-U and 2’F cubes was 8.3 nM. The buffer contained 0.2 M KCl, 10 mM HEPES, and 2 mM MgCl_2_ at pH 7.5. Nomenclature of NANP variants: DNA–DNA cubes; PS–DNA cubes with backbone phosphorothioate modifications; D-U–DNA cubes containing ssUs at the corners; 5′OH–RNA cubes with a hydroxyl group at the 5′ end; RNA–RNA cubes with a 5′-triphosphate group. The remaining RNA cubes carried 5′-triphosphates and ribose modifications, where the 2′OH was substituted with either fluorine (2′F) or a 2′-O-methyl group (2′OMe), or uridine was substituted with pseudouridine (*ψ*). In (b), each bar represents mean ± SD (*n* = 3), whereas in (c) each bar shows the mean response ± SEM (*n* = 3).

All RNA strands were synthesized via in vitro run-off transcription using either standard T7 RNA polymerase (RNAP) or a mutant T7 RNAP optimized for the incorporation of modified rNTPs [67], except for RNA cubes with 5′OH groups, which were synthesized by a commercial supplier. Interestingly, despite the successful synthesis of RNA strands containing all 2’F ribose modifications, assembly of the cubic NANPs failed, most probably by alteration of secondary structures or unpredicted interactions (data not shown). A similar situation was observed for strands containing full phosphorothioate modifications (not shown). The assembly was, however, achieved with strands containing 2’ ribose-modified pyrimidines, as shown by atomic force microscopy (AFM) and native polyacrylamide gel electrophoresis (native-PAGE) (Figure 2a). Similarly, we successfully assembled DNA cubes with backbone modifications only when the phosphorothioate bond was linked to thymidine or both cytidine and thymidine. This result suggests that even simple 3D structures can tolerate only a certain degree of modifications. Naturally, eukaryotic tRNAs contain the highest percentage (~20%) and diversity (~50 unique modifications) of modified nucleotides [68].

Incorporation of the various chemical modifications into the constituent strands of NANPs enhanced their thermal stability and increased the melting temperature as measured via thermal gradient gel electrophoresis (TGGE) (Figure 2b). While DNA cubes, regardless of modification, started to melt below 40°C, the modification of RNA cubes increased the melting temperature from 55°C to 62°C.

In addition, chemical modifications of RNA NANPs decrease sensitivity to nucleases present in fetal bovine serum for 1 h. The incorporation of 2’F, 2’OMe, and *ψ* modifications slowed the total degradation of respective RNA cubes (Figure 2c). Degradation of DNA cubes and DNA cubes having stretches of Us within corners (D-U), proceeded more slowly than RNA cubes, but the presence of uracil in DNA sequences allowed for quicker degradation when compared to unmodified DNAs.

### 3.3 | Backbone Modifications Define the Deformability of NANPs

We analyzed four different cubes separately with a single 10.2 nm diameter nanopore at 1 V to understand how their behavior changes under electrophoretic force while passing through a pore smaller than their hydrodynamic diameter [69]. Figure 2d shows ion current traces, fractional current blockade (ΔI/I_o_) vs. dwell time scatter plots, as well as dwell time distribution plots for each of the measured NANPs (from left to right, respectively). Shallow events with fractional current blockades of less than 20% were omitted. Current blockade distribution by those cubes is similar, indicating that all NANPs reach the same pore depth. However, the cubes exhibit different dwell time distributions. DNA cubes exhibit the shortest dwell time distribution, while RNA cubes exhibit the longest dwell time distribution. To further analyze these variations, we fitted the histograms using multigaussian model. The first peak (log_10_(dwell time(μs)) = 0.9 for all cubes) might correspond to collision events, second peak (log_10_(dwell time(μs)) = 1.6 for DNA, 1.3 for RNA, 1.4 for D-U and 1.4 for 2’F RNA) might correspond to partially assembled structures, while the last peak (log_10_(dwell time(μs) = 2.3 for DNA, 2.8 for RNA, 2.3 for D-U, and 2.7 for 2’F RNA) might represent correctly assembled cubes. Based on the information from the last two peaks, the fractions of correctly assembled cubes were determined to be 41% for DNA, 63% for RNA, 55% for D-U, and 66% for 2’F cubes. These values represent conservative lower limits, as the applied voltage during analysis can partially disrupt the cube structures. Therefore, the actual fraction of correctly assembled NANPs in each sample is higher than the reported values. Since all four cubes are assembled from the same number of nucleotides and share an equivalent base sequence, the observed differences in the dwell time distribution shapes suggest a variation in deformability when responding to the electrophoretic force, depending on the composition of the cubes.

### 3.4 | NANPs Differentially Activate Immune Cell Subsets Based on Their Composition

To get insight into the NANP’s effects on individual subsets of immune cells in PBMC cultures, we treated PBMCs from healthy donors with controls or representative NANPs for 24 h and analyzed cell viability and surface marker expression using multicolor flow cytometry. The representative NANPs included unmodified RNA and DNA cubes. This selection was made based on our prior studies collectively demonstrating reproducible and consistent structure-dependent immunostimulation by these materials in cultures of over 100 human donors [12–14, 16, 24, 62, 70]. LPS, ODN2216, PHA-M, and PMA/ionophore were used as positive controls due to their known ability to activate distinct subsets of immune cells present in PBMCs [24, 71–73]. Due to the breadth of immune cell subsets in PBMC and associated surface markers, we employed two antibody panels for the immunophenotyping. The first panel enumerated viable cells in various lymphocyte populations, including B cells and T cells (CD8+ T cells, CD4+ T cells, regulatory T cells (Treg), naïve T cells, and *γδ* TCR T cells) and determined cellular CD25 and CD154 expression as the markers of lymphocyte proliferation and co-stimulation/presentation, respectively (Figure S3a). The second panel determined the number of viable cells among CD14+ monocytes, plasmacytoid and myeloid dendritic cells (pDCs and mDCs, respectively), natural killer (NK) cells, and natural killer T (NKT) cells and assessed the expression of CD69 and CD54 on these cells as the markers of early activation and adhesion, respectively (Figure S3b).

**FIGURE 3 |:**
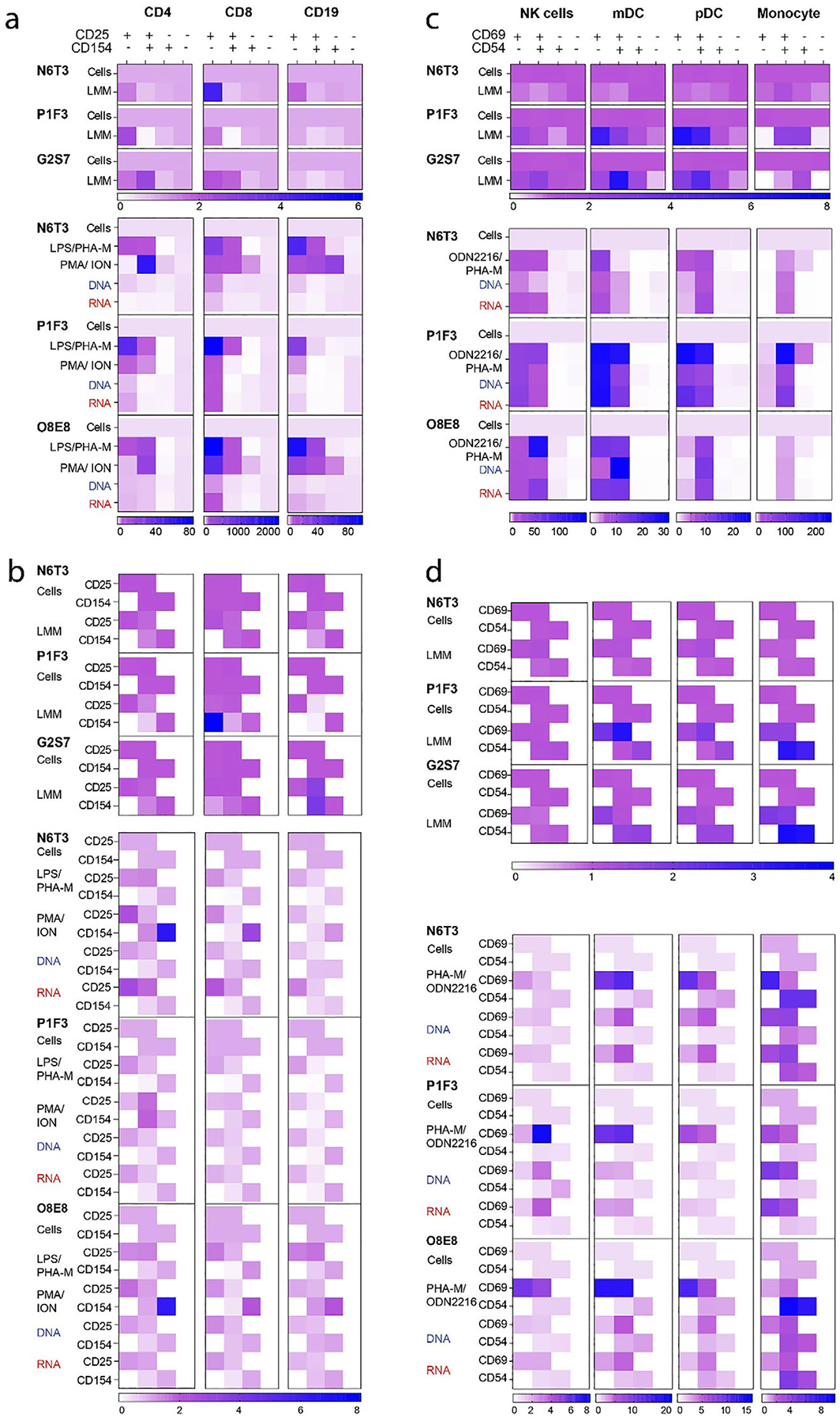
NANPs effects on the number and activation status of various immune cell subsets. Immunophenotyping panel 1 (a and b) and panel 2 (c and d) results are summarized. The samples included negative controls (untreated cells), vehicle control (LMM), positive controls (LPS/PHA-M, PMA/Ionomycin, and PHA-M/ODN2216), and NANP-treated samples (10 nM DNA cubes; 10 nM RNA cubes). The stimulation indices were calculated by comparing the treatment to the negative control. Each box in the heat map represents the average of two values for each treatment/cell population combination (*n* = 2). Data obtained from individual donor (N6T3, P1F3, G2S7, and O8E8) cultures are presented side by side. Heat maps of stimulation indices of quadrant percentages (a) and geometric mean fluorescence intensities (gMFI) (b) showing changes in CD25 and CD154 expression for each of the different cell populations—CD19+ (B cells), CD8+ (Cytotoxic T cells), and CD4+ T cells. Stimulation indices of quadrant percentages (c) and gMFI (d) showing changes in CD69 and CD54 expression for each of the different cell populations—monocytes (CD14+), pDCs (CD123+), mDCs (cD11c+), and NK cells (CD3-CD56+). Each square in the heatmaps shows a mean response of two replicates. LPS, lipopolysaccharide; PHA-M, phytohemagglutinin M; CD, cluster of differentiation; NK, natural killer; DC, dendritic cells; pDC, plasmacytoid DCs; mDCs, myeloid DCs; ION, ionophore; ODN2216, oligodeoxynucleotide 2216. Nomenclature of cube variants: DNA, DNA cubes; RNA, RNA cubes with triphosphate group on 5’ end. Assessment of physiological significance by comparing stimulation indices of individual markers is summarized in Figure S3d,e for panel 1 and Figure S3f,g for panel 2.

Both DNA and RNA cubes increased the percentage of B cells and a very small number (<0.5%) of cytotoxic—CD45RA CD8+—T cells in PBMC cultures of 2 out of the 3 donors (Figure S3c). RNA cubes were more potent than DNA cubes in increasing the percentage of CD25+/CD154− CD8+ T cells (Figure 3a,b). The greater potency of RNA cubes compared to DNA cubes observed in this analysis was not surprising and is consistent with our prior studies investigating the interferon response in PBMCs to these particles [13]. A similar structure-activity relationship (SAR) was observed in CD4+ helper T cells, where RNA cubes and DNA cubes increased the percentage of CD25+/CD154− CD4+ T cells in at least 2 out of 3 donors (Figure 3a,b).

RNA cubes, as well as DNA cubes, had comparable effects on the percentages of CD69+/CD54+ monocytes, which were higher in treated cultures compared to the control (Figure 3c,d). All NANPs resulted in an increase in CD69 and CD54 gMFI in the monocyte population (Figure 3c,d). DNA cubes increased pDC population in cultures of 2 out of 3 donors (Figure S3c). All NANPs increased the percentage of CD69+/CD54+ and CD69+/CD54− pDCs and increased CD69 gMFI in pDCs in cultures of at least 2 out of 3 donors (Figure 3c,d). While all NANPs increased mDC population, RNA cubes had the most profound effects on the mDC population, whereas DNA cubes had a weaker effect (Figure S3c). All NANPs increased the percentage of CD69+/CD54+ and CD69+/CD54− mDCs and increased CD69 gMFI in mDCs in cultures of at least 2 out of 3 donors (Figure 3c,d). RNA cubes were stronger in increasing the NK cell population in cultures of 2 out of 3 donors (Figure S3c). All NANPs increased the percentage of CD69+/CD54+ and CD69+/CD54− NK cells and increased CD69 gMFI in NK cells in cultures of at least 2 out of 3 donors (Figure 3c). RNA cubes increased the NKT cell population in all donors (Figure S3c). Stimulation indices were calculated for each cell population and analyzed markers to assess the physiological significance of the observed changes (Figure S3d,e for panel 1 and Figure S3f,g for panel 2), where a physiologically significant change is considered at least two-fold above the baseline. All changes mentioned above are physiologically significant.

Collectively, these data suggest that NANPs may enhance the effector function of T cell populations, as measured by the expression of the CD25 activation marker, induce B cell proliferation and activation, as assessed by CD154 expression, and increase both the number and early activation of innate cell populations in PBMCs. A decrease in the monocyte population observed with NANPs may be attributed to the differentiation of these cells into dendritic cells. However, the immunophenotyping experiment cannot accurately track such differentiation. The activation of innate immune cells was not unexpected, considering our previous studies that demonstrated robust and structure-dependent effects of NANPs on the activation of these cells, as monitored by the production of interferon biomarkers [13].

### 3.5 | Sugar-, Base-, and Backbone Modifications of Cube NANPs do not Trigger IFN Expression

**FIGURE 4 |:**
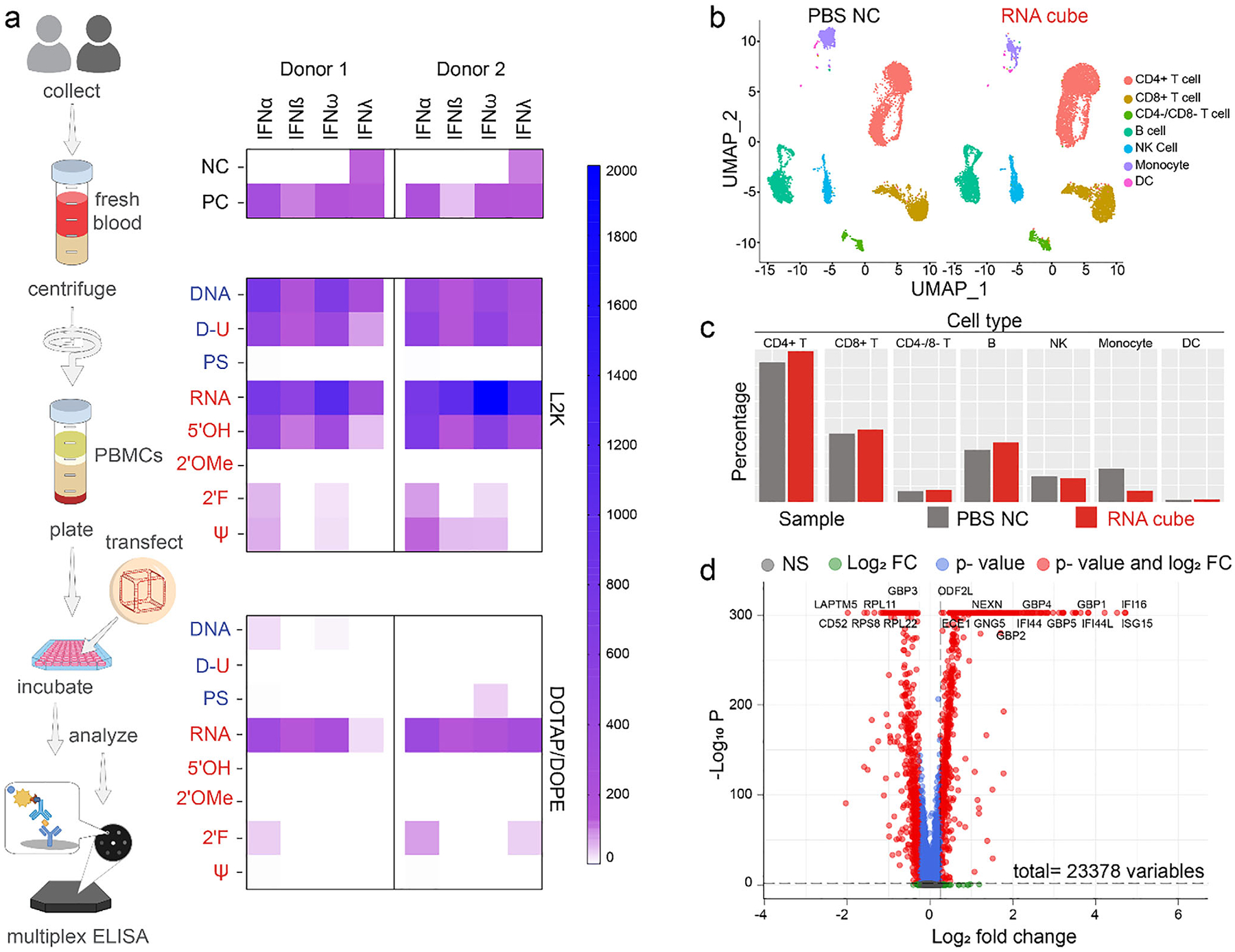
Type I and III IFN response in freshly isolated PBMCs treated with a spectrum of modified NANPs. (a) Analysis of IFN stimulation after transfection of chemically modified cube NANPs with either L2K or DOTAP/DOPE. The upper panel depicts controls, while the lower panels show stimulation after delivery of NANPs. NC, negative control (PBS), PC, positive control (mix of LPS, ODN2216, and PHA-M). Each square in the heatmap shows a mean response of two replicates. (b-d) Single-cell sequencing of PBMC treated with either PBS as a negative control (NC) or RNA cubes for 24 h before cell capture and sequencing. The cell type compositions and proportion of each cell type are displayed on the UMAP (b) and bar chart (c). The volcano plot (d) shows the differentially expressed genes in a CD4+ T cell subcluster, which has significantly more cells in the PBMC exposed to RNA cubes than in the NC. UMAP—Uniform Manifold Approximation and Projection; PBMCs, peripheral blood mononuclear cells; LPS, lipopolysaccharide; ODN2216, oligodeoxynucleotide 2216; PHA-M, phytohemagglutinin M; IFN, interferon; DOTAP – 2-dioleoyl-3-trimethylammoniumpropane; DOPE −1,2-Dioleoyl-sn-glycero-3-phosphoethanolamine; CD, cluster of differentiation; NK, natural killer; DC, dendritic cell; ELISA, enzyme-linked immunosorbent assay; PBS, phosphate-buffered saline; FC, fold change. Nomenclature of cube variants: DNA, DNA cubes; PS, DNA cubes with backbone phosphorothioate modifications; D-U, DNA cubes containing ssUs at the corners; 5′OH, RNA cubes with a hydroxyl group at the 5′ end; RNA, RNA cubes with a 5′-triphosphate group. The remaining RNA cubes carried 5′-triphosphates and ribose modifications, where the 2′OH was substituted with either fluorine (2′F) or a 2′-O-methyl group (2′OMe), or uridine was substituted with pseudouridine (*ψ*).

To gain further insight into the role of NANPs’ structural variations in their immunostimulatory properties, and particularly, their ability to trigger interferon responses, we exposed primary donor cells to cubes complexed with either L2K or DOTAP carriers for 24 h and analyzed culture supernatants by ELISA for the presence of type I and III IFNs (Figure 4a) [24]. The highest stimulation of IFNs was observed after transfection of PBMCs with L2K-complexed 5’PPP RNA cubes. The 5’PPP-containing RNA cubes stimulated the expression of all three members of type I interferon (IFN*α*, IFN*β*, IFN*ω*) and type III interferon (IFN*λ*) (Figure 4a and Figure S4). Interestingly, 5’OH RNA cubes increased the production of two type I IFNs (IFN*α* and IFN*ω*). Notable expression of IFN*α* and IFN*ω* was also observed in DNA and D-U NANP-treated cultures. RNA cubes with 2’F and *ψ*-U modifications showed significantly reduced stimulation of IFN*α* and IFN*ω* in both donors in comparison to their unmodified counterparts. On the other hand, cubes with DNA backbone modification to PS and RNA to contain 2’OMe did not elicit any interferon response, in agreement with earlier studies on traditional DNA and RNA constructs attempted for therapeutic purposes (e.g., antisense DNA oligonucleotides, siRNA, mRNA, and sgRNA) [74–76]. Type I IFNs are produced by myeloid cells, mainly monocytes and dendritic cells, in response to a viral infection. These data are consistent with our earlier study [13] demonstrating that RNA and DNA NANPs stimulate interferon response similar to that observed during viral infections and demonstrate that composition and chemical modifications allow tuning of the magnitude of the interferon response to these nanoparticles.

NANPs complexation with DOTAP/DOPE resulted in significantly reduced IFN response to all cubes (Figure 4a and Figure S4), where D-U, 5’OH, and *ψ* cubes did not activate IFN at least in one donor. Only 5’PPP-containing RNA cubes activated all three members of type I IFN (IFN-*α*, IFN-*β*, IFN-*ω*) and type III IFN (IFN-*λ*) in both donors. The 2’F RNA cubes stimulated IFN*α* in both donors and IFN*λ* in one donor.

Endosomal TLRs are differentially expressed in various immune cell populations present in blood and PBMC [77, 78]. TLR7 and TLR8 have non-redundant activities and differentially activate IRF and NFk*β* pathways [78]. For example, pDCs and B cells express TLR7, the primary receptor for NANPs-mediated type I/III IFN response [12]. T-cells express IFN*α* receptors (R1 and R2), and B cells express IFN*α*R1 and IFN*λ* receptors. The IFNs produced by pDCs and mDCs can activate T and B cells via these receptors. NANPs are associated with scavenger receptors and are internalized into endosomes, where they can interact with TLR7 to initiate the IFN response. Type I IFN (IFN-*α*, IFN-*β*, IFN-*ω*) activates the IRF transcription factors, which commonly lead to antiviral activities such as the inhibition of translation, mRNA degradation, and inhibition of virus transcription and assembly. Type I IFNs also activate NK cells, which provide rapid responses to virus-infected cells and other intracellular pathogens; they can inhibit the production of certain proinflammatory cytokines in T cells. Finally, type III IFN (IFN-*λ*) upregulates the expression of genes controlling viral replication and host cell proliferation. Therefore, in conjunction with the immunophenotyping data demonstrating the differential effects of NANPs on various immune cell populations present in PBMCs (Figure 3), IFN responses detected in supernatants (Figure 4) reflect the complexity of both direct (PRR/TLR-mediated) and indirect (cytokine-mediated) effects of NANPs on cells.

### 3.6 | RNA Cubes Elicit Responses Similar to Those Induced by Viral Infection

To elucidate molecular determinants of the NANPs-mediated activation of immune cells, we assessed single-cell transcriptomes in control versus NANPs-treated cells. For this experiment, we selected the PBMCs of one donor (P1F3), whose cultures in the immunophenotyping experiment (Figure 3) demonstrated the most robust effects, and RNA cubes as the representative NANPs with the most robust immunostimulatory activity. Single-cell sequencing captured cells from several populations, including CD4+ T cells, CD8+ T cells, CD4−/CD8− T cells, B cells, NK cells, monocytes, and dendritic cells (Figure 4b). An increase in the number of cells of all populations was seen in the RNA cubes-exposed cultures vs. control cells, except for NK cells and monocytes, whose numbers were lower in the treatment group (Figure 4c). A statistically significant decrease in the number of cells was detected only in the monocyte population (Figure S5), which may suggest their differentiation to dendritic cells and/or exhaustion and is consistent with the immunophenotyping data (Figure 3). A total of 1565 differentially expressed genes were detected in RNA-cube-treated cells (Figure 4d). Examples of differential expressions of genes observed across all cell populations include ISG15, IFI6, CXCL10, CD52, LYZ, and S100A4 (Figure S6). The top twenty pathways statistically significantly affected by the RNA cube exposure included pathways involved in interferon signaling and cellular responses to a virus (Figure S7). These data are in agreement with the immunophenotyping and cytokine responses observed in this study and with our earlier studies of RNA cubes [13], and suggest that immune cells in PBMCs respond to RNA cubes in a similar way to how they respond to a viral infection.

### 3.7 | Identifying PRRs Involved in NANP Recognition Using Reporter Cell Lines

**FIGURE 5 |:**
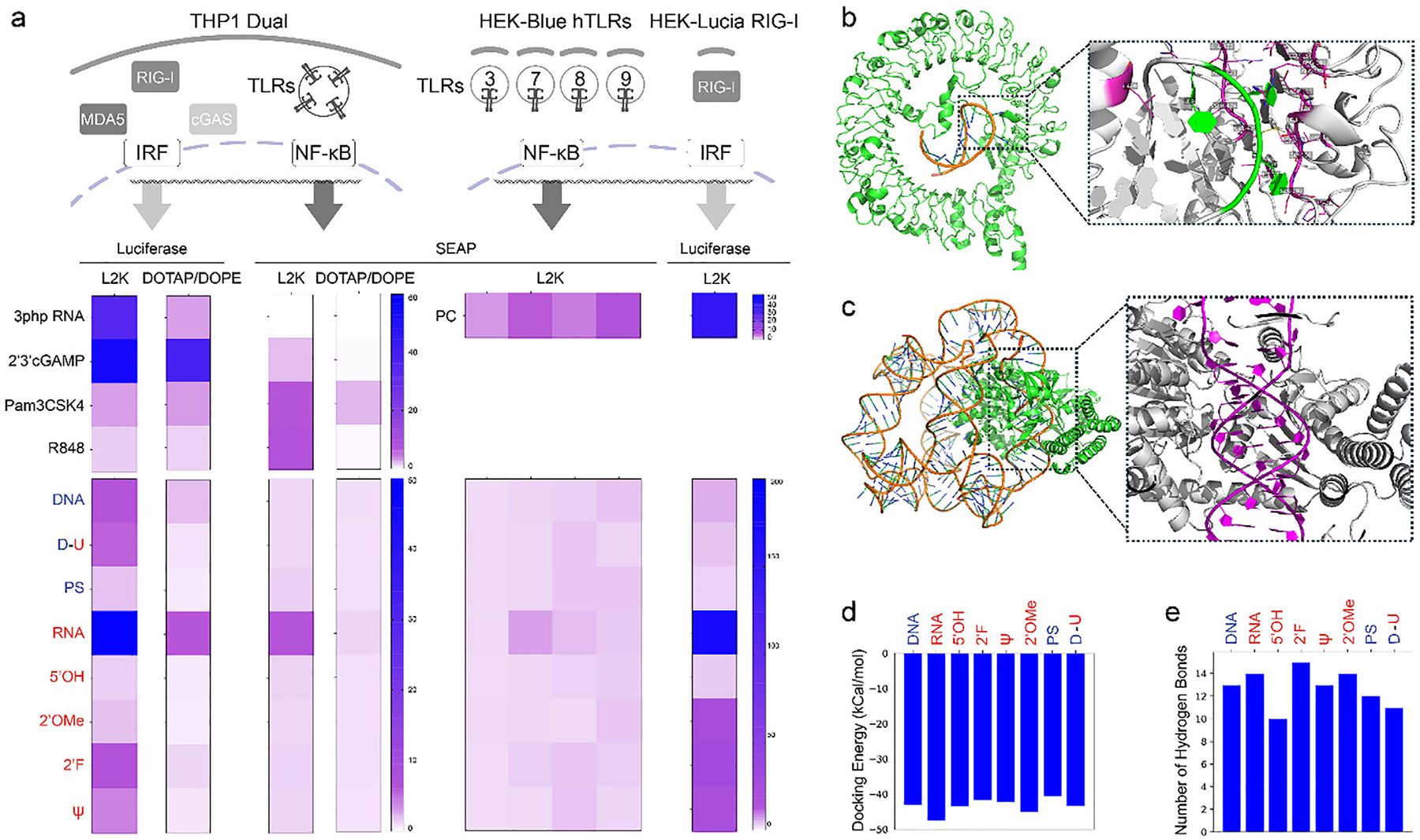
Mechanistic studies identifying pattern recognition receptors (PRRs) involved in NANP recognition, combined with computational modeling of the interplay between selected chemical modifications and their corresponding PRRs. (a) Activation of sensing mechanisms in reporter cell lines and schematics of their activation through IRF or NF-*κ*B transcription factors after delivery of NANPs by L2K or DOTAP/DOPE carriers. Left side-converging of activation either to IRF or NF-*κ*B transcription factor families by cube NANPs in THP1 Dual cells. Right side-Activation of cytosolic (RIG-I) or endosomal (TLRs) sensing mechanisms in engineered HEK cell lines. Stimulation of NF-*κ*B transcription factors through TLRs in individual reporter cell lines after delivery of NANPs by L2K carrier. Transfection of HEK-Lucia RIG-I cells by L2K strongly stimulated RIG-I by in vitro synthesized RNA cubes, while synthetic RNA cubes are immunoquiescent. The introduction of 2’F, 2’OME, and *ψ* RNA significantly decreases the activity of corresponding NANP cubes. Activation of TLRs 3, 7, 8, and 9 by RNA cubes is minimal. (b) Docking structure of TLR7 with a fragment (GGCAACUUUGAUCCC) from the RNA cube. (c) Docking structure of RIG-I with the RNA cube. (d) MedusaScore energy for the interaction between the UUU region of the cube and TLR7. (e) Average number of hydrogen bonds formed between the cube structure and RIG-I during molecular dynamics simulations. TLRs, toll-like receptors; RIG-I, retinoic acid inducible gene; Pam3CSK4, synthetic lipopeptide Pam3CysSerLys4, where Pam3 is the three palmitoyl groups; Cys is cysteine, Ser is serine, and Lys4 is 4 lysines; cGAMP, cyclic guanosine monophosphate-adenosine monophosphate. Nomenclature of cube variants: DNA, DNA cubes; PS, DNA cubes with backbone phosphorothioate modifications; D-U, DNA cubes containing ssUs at the corners; 5′OH, RNA cubes with a hydroxyl group at the 5′ end; RNA, RNA cubes with a 5′-triphosphate group. The remaining RNA cubes carried 5′-triphosphates and ribose modifications, where the 2′OH was substituted with either fluorine (2′F) or a 2′-O-methyl group (2′OMe), or uridine was substituted with pseudouridine (*ψ*).

Next, we sought to explore PRRs primarily activated by NANPs with different compositions and chemical modifications. The models employed included those specific to the Nuclear Factor kappa light chain enhancer of activated B cells (NF-*κ*B) or interferon-regulatory factor (IRF) transcription factors. THP1-Dual is a monocyte-derived cell line engineered to express secreted alkaline phosphatase (SEAP) when triggered pathways merge to NF-*κ*B, or luciferase when pathways activate IRF (Figure 5a and Figure S8a). We observed the most robust luciferase response after transfection of 5’-PPP-containing RNA cubes with L2K. The incorporation of 2’F, 2’OMe, or *ψ*’s significantly decreased the activation of the IRF pathway. A notable increase in activation was also observed for DNA cubes. Activation for 5’PPP RNA cubes and slightly for DNA cubes was also observed in cultures that utilized the DOTAP/DOPE carrier for NANPs delivery onto the cells. The SEAP production promoted by NF-*κ*B was negligible for all assemblies except RNA cubes, regardless of carrier.

The observed activation that resulted in IRF-driven expression of luciferase directed us to focus on the RIG-I, the main cytosolic receptor of RNA, while comparing it to relevant TLR receptors in HEK-Lucia RIG-I or HEK-Blue TLR engineered reporter cell lines, respectively (Figure 5a and Figure S9).

RIG-I-like receptors (RIG-I, MDA5, and LGP2) monitor for RNA of infectious agents released from endosomes or bypassing the endosome route. RIG-I is a crucial part of an innate cellular defense that distinguishes foreign RNAs from cellular transcripts. The presence of 5’PPP (on the 5’-blunt end at least 10-nucleotidelong RNA duplexes most effectively activates RIG-I downstream signaling, though even ssRNA with 5’PPP may trigger RIG-I-mediated response [79].

RIG-I detects 5’PPP and 5’PP of RNAs. Except for synthetic RNA-based cubes, all our in vitro transcribed RNA cubes contain six 5’PPPs and, as such, can be immunostimulatory via the RIG-I mediated pathway. This was confirmed by the HEK-Lucia RIG-I cell line, where nonmodified RNA cubes containing 5’PPP produced a strong response; however, modified RNA cubes, albeit containing 5’PPP, produced significantly lower responses. As predicted, synthetic RNA cubes that contain six 5’OH groups did not activate RIG-I. Interestingly, we haven’t seen a similar response after transfection of NANPs by DOTAP/DOPE (not shown).

To further dissect possible interactions with endosome-residing PRRs, we screened HEK-Blue cells expressing the given TLRs, whose activation leads to SEAP production. In surveyed TLR3 (sensing dsRNA), TLR7/8 (ssRNA), and TLR9 (unmethylated CpG DNA), we found that only RNA cubes containing 5’PPP groups were able to activate TLR7 and, to a lesser extent, TLR8, whereas other chemically modified NANPs’ stimulation was below two-fold induction over untreated cells, considered, thus, pharmacologically insignificant. Modified RNA-based NANPs (2’F, *ψ*) resulted in a weak activation of TLR8, and 2’OMe cubes weakly activated TLR9; however, it was not significantly different than SEAP production measured in the carrier alone control. Neither TLR3 nor TLR7 was able to recognize any of the chemically modified NANPs (Figure 5a and Figure S9).

We then investigated the possibility of tuning the immunorecognition of cubes further by mixing different chemically modified strands into their composition. Incorporation of 2′F monomers into RNA cube assemblies lowered IRF signaling in THP-1 Dual cells and HEK-Lucia RIG-I cells (Figure S8b). Although the ratio of 2′F to transcribed RNA strands was gradually increased from 5:1 to 6:0, no consistent overall pattern was observed. Interestingly, embedding just a single 2′F strand within an RNA cube reduced its immunostimulatory potential by more than half, and the second-highest IRF activation was observed at a 4:2 ratio of 2′F to RNA strands. A comparison of various hybrid cubes with balanced 3:3 ratios of modified to unmodified 5’PPP RNA strands revealed that 5′OH:RNA cubes showed the highest immunostimulatory potential, whereas 2′F:RNA cubes had the lowest. DNA:RNA, *ψ*:RNA, and D-U:RNA hybrids displayed similar levels of activity (Figure S8b). In comparison, we can observe a gradual decrease of immunostimulatory potential with increasing ratio of 2’F monomers in cube NANPs after transfection to HEK-Lucia RIG-I cells (Figure S8b). However, except for substitution with DNA strands, replacing three RNA monomers with 5′OH, *ψ*, and D-U strands has not decreased immunostimulatory potential. This approach offers an interesting avenue for the further personalization of NANPs for specific immunological applications. More detailed studies, however, will be required to determine how the position of specific modifications within cube assemblies, as well as changes in their chemical and thermodynamic stabilities, influence immunorecognition.

### 3.8 | Computational Models Suggest NANP-PRR Interactions

We initially constructed complex structures between various RNA cubes and TLR7 or RIG-I using the AlphaFold3 webserver [80]. AlphaFold3 was utilized specifically to generate the initial structures of the RNA cube-protein complexes. Afterward, we modified the RNA cube to represent the seven other cube variants for further analysis. While AlphaFold3 successfully predicted a reasonable docking structure for the RIG-I/RNA cube complex, it was unable to produce a reliable docking model for the TLR7/RNA cube. To address this, we tested several RNA cube fragments to observe how each interacted with TLR7. Regardless of the fragment used, we consistently found that the “UUU” region (or “TTT” in DNA) was responsible for interacting with TLR7 (Figure 5b). Based on these findings, we extracted the “UUU” fragment from different RNA cubes and performed docking simulations with TLR7 using MedusaDock [40–42]. The results indicated that RNA cubes with 5’ triphosphates exhibited the lowest docking energy [81] (Figure 5d), suggesting that 5’ triphosphate modification enhances the binding affinity between the cube and TLR7. For the RIG-I complex, AlphaFold3 generated a reliable docking structure, as illustrated in Figure 5c, which served as the basis for subsequent molecular dynamics simulations. Throughout the simulations, we calculated the average number of hydrogen bonds formed between the RNA cube and RIG-I. Our analysis revealed that the original RNA cube, the DNA cube with phosphorothioated thymidines, and DNA cubes with uridines at their corners exhibited fewer hydrogen bonds with RIG-I (Figure 5e), suggesting weaker binding affinities compared to other cube structures.

### 3.9 | RNA Cubes Contribute to Vaccine Efficacy Without Eliciting Autoimmunity In Vivo

**FIGURE 6 |:**
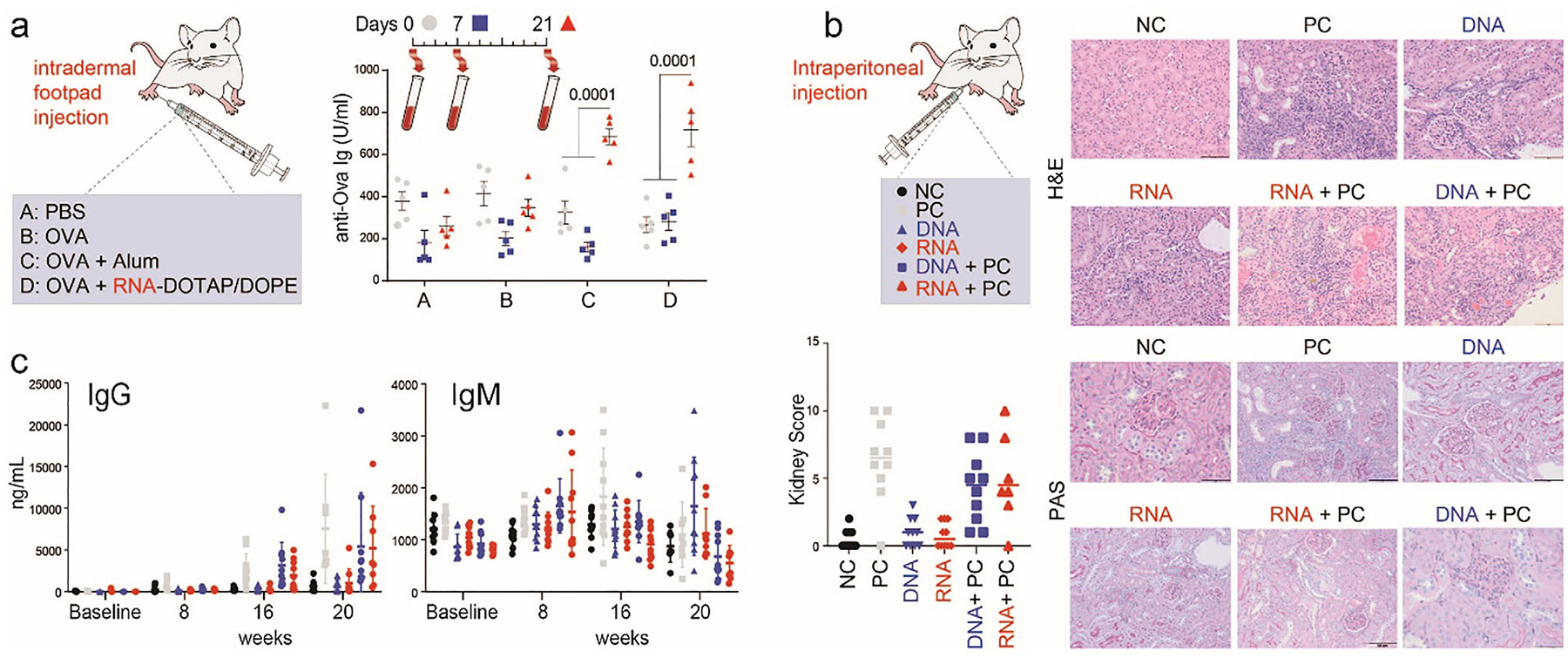
RNA cubes as safe adjuvant in vivo. (a) Adjuvant activity of RNA cubes complexed with DOTAP/DOPE. The OVA was administered intradermally using rear footpad injection either alone or in the presence of Alum adjuvant or RNA cubes; the levels of OVA-specific antibodies were monitored by ELISA before immunization, 7, and 21 days after the immunization. Each data point represents the score of an individual animal, and bars indicate the group mean ± SEM (*n* = 5) (b) Assessing the risk of autoimmunity. Eight-week-old female SJL/J mice were administered a single *i.p*. dose of PBS as a negative control (NC), pristane as a positive control (PC), 10 nM RNA cubes or DNA cubes alone (RNA cubes; DNA cubes), or in combination with pristane (RNA cubes + PC; DNA cubes + PC). The blood was collected before the injection and after 8-, 16-, and 20-weeks postinjection. At the end of the study, on week 20, the animals were sacrificed, and 10% NBF-fixed kidney tissues were stained by H&E or PAS for subsequent histological analysis. The total kidney score represents the total degree of kidney tissue damage at week 20. Representative kidney tissues after H&E or PAS staining. Each data point represents the score of an individual animal, and bars indicate the group median ± SD (*n* = 10). (c) Blood levels of anti-dsDNA IgG and IgM at various time points. Each data point represents the mean response of two replicates from an individual animal, and bars indicate the group mean ± SD (*n* = 10). Statistical significance was assessed using ordinary one-way ANOVA, with *p* values indicated. OVA – ovalbumin; H&E, hematoxylin and eosin; PAS, Periodic Acid-Schiff; PC, positive control; NC, negative control; Ig, immunoglobulin; PBS, phosphate-buffered saline. Nomenclature of cubes used: DNA, DNA cubes; RNA, RNA cubes with triphosphate group on 5’ end.

Since the in vitro data described above collectively suggested a potential utility of RNA cubes as an adjuvant, we investigated the ability of RNA cubes to enhance the immunogenicity of a model antigen; the standard-of-care adjuvant Alum was used as a positive control. Since the extensive dataset, including our earlier studies [62, 70] and the data presented above, demonstrated that beneficial induction of type I and type III interferons by NANPs is only possible after NANPs complexation with lipid-based carriers [70], and different lipofectamines used in our in vitro experiments are not suitable for in vivo use, RNA cubes were complexed with animal-friendly lipid DOTAP/DOPE, which was confirmed in vitro to retain NANPs’ ability to induce interferon response following complexation (Figure 4 and Figure S4). In this study, the RNA cube–DOTAP/DOPE complex is considered as a single product and analyzed as one entity. As a model antigen, we used endotoxin-free, vaccine-grade ovalbumin (OVA). The OVA was administered intradermally using a rear footpad injection, either alone or in the presence of Alum adjuvant or RNA cubes-DOTAP/DOPE complex. Since complexation of NANPs with a lipid-based carrier neutralizes the carrier’s cationic groups, testing free cationic lipid with OVA would not serve as an appropriate control. Therefore, the DOTAP/DOPE–OVA mixture was not tested. The primary role of the DOTAP/DOPE formulation is to facilitate intracellular delivery of RNA cubes to induce type I/III interferons. The levels of OVA-specific antibodies were monitored by ELISA before immunization, at 7 and 21 days after a single footpad immunization according to the protocol established earlier for nanoparticle-based vaccine [39]. Although the level of antibodies 7 days post-injection did not increase, after 21 days, the level of antibodies was significantly higher than at 7 days for both groups of mice immunized with Alum/OVA and RNA cubes/DOTAP/DOPE/OVA (Figure 6a). Administration of the RNA cubes complexed with DOTAP/DOPE had a similar adjuvant effect as Alum.

Traditional TLR agonists, such as LPS, resiquimod, imiquimod, and CpG DNA, have been explored for cancer therapy and vaccine adjuvants. However, their broad, potent immunostimulatory activities, leading to overt immunostimulation upon systemic administration, and the induction of autoimmunity even after local administration, tremendously limited their clinical applications [82–84]. At the moment, traditional TLR agonists safely used in clinical applications include either those administered topically (e.g., TLR7 agonist imiquimod in Aldara cream) or modified to reduce their potency (e.g., TLR4 agonist monophosphoryl lipid A in FENDrix and Cervarix products that are Hepatitis B and HPV vaccines, respectively) (package inserts can be searched in https://open.fda.gov/fdalabels/). Therefore, it was important to study RNA cubes to understand the potential risk of macromolecular nanomaterials (NANPs) to induce autoimmunity. To answer this question, we employed the Swiss Jim Lambert (SJL/J) mouse model, which is genetically predisposed to autoimmune reactions and is commonly used to study autoimmunity [85, 86]. The study was conducted in females, representing the most sensitive model. Female SJL/J mice display greater mortality, kidney disease, and blood levels of anti-dsDNA antibodies than male mice. This is similar to the incidence of systemic lupus erythematosus in humans, where the men: women SLE incidence ratio is 1:9, and the disease severity is greater in women [87]. Chemically induced SLE in this model develops ~week 15, and by week 35, only 30% of animals are still alive [86]. Unlike pristane, used in the study as a positive control, the administration of RNA cubes did not affect animal survival, quality of life, kidney tissue, or the formation of anti-dsDNA-specific antibodies, even after five months post-exposure. Furthermore, co-administration of RNA-cubes or DNA-cubes with the positive control, pristane, did not increase pristane-mediated kidney damage or anti-dsDNA antibody levels (Figure 6b,c). These data demonstrate that NANPs, when used alone, do not increase the risk of autoimmunity, which is consistent with the prior studies demonstrating their immunoquiescent nature [13] and a similar lack of autoimmunity with DNA origami particles also explored in vaccines [88]. Together with the adjuvanticity study using a model antigen, these data expand the current knowledge base regarding various adjuvants [89]. Further research is necessary to verify how NANPs’ complexation with carriers alone and in the context of different antigens may influence their effects on the risk of autoimmunity in a predisposed host.

### 3.10 | RNA Cubes do not Disrupt Epithelial Barriers

The epithelial barrier protects the body’s internal and external surfaces, including the skin, lungs, intestines, and urinary tract. It is essential for maintaining homeostasis, defending against infections, and modulating immune responses. The 3D cell culture on-chip models are increasingly effective in simulating complex in vivo interactions as demonstrated by a blood-vessel-on-a-chip model composed of endothelial and mural cells that revealed how endothelial barrier failure, followed by inflammation, disrupted the connections between endothelial and mural cells [90]. Furthermore, Multi-organ-on-chip systems (MOOCs) can simulate vaccination by supporting lymph nodes ex vivo. This model enables the study of antigen injection into mock tissue, leading to localized antigen accumulation and corresponding changes in activation of markers and gene expression within the lymph node [91]. Therefore, we examined the influence of NANPs delivery on the barrier integrity in 3D cell cultures of Caco-2 cells, a model of the intestinal epithelial barrier. We observed that in all except untreated cells, treatment decreased the transepithelial electrical resistance (TEER) of the Caco-2 tubules after 24 h of transfection. However, after 72 h, with the exception of D-U cubes, RNA and DNA monomers complexed with L2K, and L2K alone, TEER was comparable to pretreatment values, indicating that it is L2K that might disrupt the barrier integrity of the tubules (Figure S10a).

The fluorescence imaging of the Alexa 488 labelled NANPs transfected in the OrganoPlate shows higher uptake for some of the cubes, especially when complexed with L2K as compared to DOTAP/DOPE, except for the DNA cubes. The images show no uptake of both RNA or DNA cubes monomers when complexed with L2K or DOTAP/DOPE, and no uptake from the vehicle controls.

The quantified uptake of modified cubes was processed with ImageJ, and a two-way ANOVA (*p < 0.05, ***p < 0.001) was performed to determine significance, and the data are normalized to untreated cells. The results show that after 72 h, there was no uptake for the RNA/DNA monomers complexed with either carrier, indicating that they might have been degraded by the cells at the endpoint of the experiment. As for the DNA cubes, there was a significant difference for the cubes complexed with DOTAP DOPE when compared to cubes complexed with L2K, where DOTAP/DOPE uptake was higher in this single instance. For D-U, *ψ*, and 5’OH cubes, complexing with L2K resulted in significantly higher uptake as compared to complexing to DOTAP/DOPE (Figure S10b,c).

## 4 | Conclusions

NANP technology is emerging as a promising, safe, and effective immunomodulatory tool. RNA cube NANPs have demonstrated the ability to enhance the effector function of T cell populations, induce B cell proliferation and activation, and increase the number and early activation of innate immune cell populations in PBMCs. This immunostimulatory effect mimics the body’s response to viral infections, as evidenced by interferon stimulation of expression as well as single-cell sequencing data. The activation of ISGs suggests that RNA cubes could be used to boost immune responses.

Chemical modifications to the constituent strands of RNA cubes, such as the incorporation of 2’F or 2’OMe groups, play a crucial role in enhancing thermal stability, increasing nuclease resistance, and reducing immunostimulatory potential. These modifications are essential for fine-tuning the immune response and ensuring the safety of NANPs in therapeutic applications. By controlling these properties, one can design NANPs that elicit beneficial immune responses without overstimulating the immune system, such as that needed for vaccine efficacy.

Indeed, RNA cubes complexed with appropriate delivery carriers, such as DOTAP/DOPE, exhibit adjuvant activity comparable to standard adjuvants like Alum and enhance the immunogenicity of the model antigen, as demonstrated by increased antibody levels in immunized mice. Moreover, unlike traditional adjuvants, RNA cubes do not increase the risk of autoimmunity. Studies in murine models genetically predisposed to autoimmune reactions have shown that RNA cubes do not affect animal survival, quality of life, or induce autoimmune responses, even after prolonged exposure. This finding highlights the potential of RNA cubes as a safer alternative to traditional adjuvants, which can sometimes lead to adverse effects. The delivery of RNA cube nanoparticles does not disrupt the integrity of the epithelial barrier in 3D cell culture models, indicating their potential for safe administration via various routes, including mucosal surfaces. This feature is crucial for developing vaccines and therapies that require mucosal administration, as it ensures that the epithelial barrier remains intact and functional.

The limitations of the current study include the use of lipid carriers, model antigen, and routes of administration, which may be different from those used in the lead vaccine candidate. However, these limitations do not diminish the value of NANPs characterization data presented herein. Furthermore, these data warrant additional investigation and exploration of NANPs as adjuvants for various disease-specific antigens in relevant models using disease- and model-appropriate routes and frequencies of administration.

Collectively, these data suggest that RNA cube nanoparticles represent a promising frontier in immunotherapeutic applications. Their ability to modulate immune responses safely and effectively, combined with their adjuvant activity and epithelial barrier integrity, makes them a strong candidate for further research and clinical trials. As our understanding of these nanoparticles continues to grow, so too does their potential to revolutionize the field of immunotherapy.

## Supplementary Material

SI

Additional supporting information can be found online in the Supporting Information section.

**Supporting File**: adfm73865-sup-0001-SuppMat.docx

## Data Availability

The data that support the findings of this study are available from the corresponding author upon reasonable request.
