# Supplementary material for: Expanding Chemical Space of Nucleic Acid Nanoparticles for Tunable Antiviral-Like Immunomodulatory Responses and Potent Adjuvant Activity
Source: Adv Funct Mater. Author manuscript; Available in PMC 2026 Feb 12. (PMC12893623; doi:10.1002/adfm.202515585)
Supplement: SI [file NIHMS2145099-supplement-SI.docx]

Supporting information

**Expanding Chemical Space of Nucleic Acid Nanoparticles for Tunable Antiviral-like Immunomodulatory Responses and Potent Adjuvant Activity**

Martin Panigaj^1#^, Hannah S. Newton^2#^, Jian Wang^3^, Laxmi Pandey^4^, Yelixza Avilla^1^, Phong Nguyen^1^, Morgan Chandler^1,5^, Justin Halman^1^, Yasmine Radwan^1^, Stephanie Smith^6^, Elijah Edmondson^6^, Simone Difilippantonio^7^, Chelsea Sanders^7^, Bao Tran^8^, Yongmei Zhao^9^, Shaojun Xie^9^, Edward Cedrone^2^, Barry W. Neun^2^, Meni Wanunu^4^, Nikolay V. Dokholyan^3^, Marina A. Dobrovolskaia^2^, and Kirill A. Afonin^1^*

1 – Nanoscale Science Program, Department of Chemistry, University of North Carolina at Charlotte, Charlotte, NC 28223, USA

2 – Nanotechnology Characterization Laboratory, Cancer Research Technology Program, Frederick National Laboratory for Cancer Research sponsored by the National Cancer Institute, Frederick, MD 21702, USA

3 – Department of Neurology, University of Virginia, School of Medicine, Charlottesville, VA 22903, USA

4 – Department of Physics, Department of Bioengineering, Northeastern University, Boston, MA 02115, USA

5 – MIMETAS US, Inc, Gaithersburg, MD 20878, USA

6 – Molecular Histopathology Laboratory, Laboratory of Animal Sciences Program, Frederick National Laboratory for Cancer Research sponsored by the National Cancer Institute, Frederick, MD 21702, USA.

7 – Animal Research Technical Support, Laboratory of Animal Sciences Program, Frederick National Laboratory for Cancer Research sponsored by the National Cancer Institute, Frederick, MD 21702, USA

8 – CCR Sequencing Facility, Cancer Research Technology Program, Frederick National Laboratory for Cancer Research sponsored by the National Cancer Institute, Frederick, MD 21702, USA

9 – CCR Sequencing Facility, Bioinformatics Group, Bioinformatics and Computational Science, Frederick National Laboratory for Cancer Research sponsored by the National Cancer Institute, Frederick, MD 21702, USA

# - equal contribution

*- correspondence to [kafonin@charlotte.edu](mailto:kafonin@charlotte.edu) and [marina@mail.nih.gov](file:///D:\\Kingstone%20BackUp%2002-06-25\\MP_KINGSTON\\3_Afonin%20Lab\\0_Projects\\0_Project_Chemically%20modified%20cubes\\Article\\Pre-Submision%20Edits\\marina@mail.nih.gov)

**Sequences used in this project:**

**RNA cubes with 5'PPP**

**rA**: 5'-PPP-rGrGrC rArArC rUrUrU rGrArU rCrCrC rUrCrG rGrUrU rUrArG rCrGrC rCrGrG rCrCrU rUrUrU rCrUrC rCrCrA rCrArC rUrUrU rCrArC rG -3'

**rB**: 5'-PPP-rGrGrG rArArA rUrUrU rCrGrU rGrGrU rArGrG rUrUrU rUrGrU rUrGrC rCrCrG rUrGrU rUrUrC rUrArC rGrArU rUrArC rUrUrU rGrGrU rC -3'

**rC**: 5'-PPP-rGrGrA rCrArU rUrUrU rCrGrA rGrArC rArGrC rArUrU rUrUrU rUrCrC rCrGrA rCrCrU rUrUrG rCrGrG rArUrU rGrUrA rUrUrU rUrArG rG -3'

**rD**: 5'-PPP-rGrGrC rGrCrU rUrUrU rGrArC rCrUrU rCrUrG rCrUrU rUrArU rGrUrC rCrCrC rUrArU rUrUrC rUrUrA rArUrG rArCrU rUrUrU rGrGrC rC -3'

**rE**: 5'-PPP-rGrGrG rArGrA rUrUrU rArGrU rCrArU rUrArA rGrUrU rUrUrA rCrArA rUrCrC rGrCrU rUrUrG rUrArA rUrCrG rUrArG rUrUrU rGrUrG rU -3'

**rF**: 5'-PPP-rGrGrG rArUrC rUrUrU rArCrC rUrArC rCrArC rGrUrU rUrUrG rCrUrG rUrCrU rCrGrU rUrUrG rCrArG rArArG rGrUrC rUrUrU rCrCrG rA -3'

**RNA cubes with 5’OH**

**rA**: 5'-OH-rGrGrC rArArC rUrUrU rGrArU rCrCrC rUrCrG rGrUrU rUrArG rCrGrC rCrGrG rCrCrU rUrUrU rCrUrC rCrCrA rCrArC rUrUrU rCrArC rG -3'

**rB:** 5'-OH-rGrGrG rArArA rUrUrU rCrGrU rGrGrU rArGrG rUrUrU rUrGrU rUrGrC rCrCrG rUrGrU rUrUrC rUrArC rGrArU rUrArC rUrUrU rGrGrU rC -3'

**rC**: 5'-OH-rGrGrA rCrArU rUrUrU rCrGrA rGrArC rArGrC rArUrU rUrUrU rUrCrC rCrGrA rCrCrU rUrUrG rCrGrG rArUrU rGrUrA rUrUrU rUrArG rG -3'

**rD**: 5'-OH-rGrGrC rGrCrU rUrUrU rGrArC rCrUrU rCrUrG rCrUrU rUrArU rGrUrC rCrCrC rUrArU rUrUrC rUrUrA rArUrG rArCrU rUrUrU rGrGrC rC -3'

**rE**: 5'-OH-rGrGrG rArGrA rUrUrU rArGrU rCrArU rUrArA rGrUrU rUrUrA rCrArA rUrCrC rGrCrU rUrUrG rUrArA rUrCrG rUrArG rUrUrU rGrUrG rU -3'

**rF**: 5'-OH-rGrGrG rArUrC rUrUrU rArCrC rUrArC rCrArC rGrUrU rUrUrG rCrUrG rUrCrU rCrGrU rUrUrG rCrArG rArArG rGrUrC rUrUrU rCrCrG rA -3'

**RNA cubes with 2'F pyrimidines(underlined)**

**rA**: 5'-PPP-rGrGrC rArArC rUrUrU rGrArU rCrCrC rUrCrG rGrUrU rUrArG rCrGrC rCrGrG rCrCrU rUrUrU rCrUrC rCrCrA rCrArC rUrUrU rCrArC rG -3'

**rB**: 5'-PPP-rGrGrG rArArA rUrUrU rCrGrU rGrGrU rArGrG rUrUrU rUrGrU rUrGrC rCrCrG rUrGrU rUrUrC rUrArC rGrArU rUrArC rUrUrU rGrGrU rC -3'

**rC**: 5'-PPP-rGrGrA rCrArU rUrUrU rCrGrA rGrArC rArGrC rArUrU rUrUrU rUrCrC rCrGrA rCrCrU rUrUrG rCrGrG rArUrU rGrUrA rUrUrU rUrArG rG -3'

**rD**: 5'-PPP-rGrGrC rGrCrU rUrUrU rGrArC rCrUrU rCrUrG rCrUrU rUrArU rGrUrC rCrCrC rUrArU rUrUrC rUrUrA rArUrG rArCrU rUrUrU rGrGrC rC -3'

**rE**: 5'-PPP-rGrGrG rArGrA rUrUrU rArGrU rCrArU rUrArA rGrUrU rUrUrA rCrArA rUrCrC rGrCrU rUrUrG rUrArA rUrCrG rUrArG rUrUrU rGrUrG rU -3'

**rF**: 5'-PPP-rGrGrG rArUrC rUrUrU rArCrC rUrArC rCrArC rGrUrU rUrUrG rCrUrG rUrCrU rCrGrU rUrUrG rCrArG rArArG rGrUrC rUrUrU rCrCrG rA -3'

**RNA cubes with 2’OMe pyrimidines(underlined)**

**rA**: 5'-PPP-rGrGrC rArArC rUrUrU rGrArU rCrCrC rUrCrG rGrUrU rUrArG rCrGrC rCrGrG rCrCrU rUrUrU rCrUrC rCrCrA rCrArC rUrUrU rCrArC rG -3'

**rB**: 5'-PPP-rGrGrG rArArA rUrUrU rCrGrU rGrGrU rArGrG rUrUrU rUrGrU rUrGrC rCrCrG rUrGrU rUrUrC rUrArC rGrArU rUrArC rUrUrU rGrGrU rC -3'

**rC**: 5'-PPP-rGrGrA rCrArU rUrUrU rCrGrA rGrArC rArGrC rArUrU rUrUrU rUrCrC rCrGrA rCrCrU rUrUrG rCrGrG rArUrU rGrUrA rUrUrU rUrArG rG -3'

**rD**: 5'-PPP-rGrGrC rGrCrU rUrUrU rGrArC rCrUrU rCrUrG rCrUrU rUrArU rGrUrC rCrCrC rUrArU rUrUrC rUrUrA rArUrG rArCrU rUrUrU rGrGrC rC -3'

**rE**: 5'-PPP-rGrGrG rArGrA rUrUrU rArGrU rCrArU rUrArA rGrUrU rUrUrA rCrArA rUrCrC rGrCrU rUrUrG rUrArA rUrCrG rUrArG rUrUrU rGrUrG rU -3'

**rF**: 5'-PPP-rGrGrG rArUrC rUrUrU rArCrC rUrArC rCrArC rGrUrU rUrUrG rCrUrG rUrCrU rCrGrU rUrUrG rCrArG rArArG rGrUrC rUrUrU rCrCrG rA -3'

**RNA cubes with ψ (rψ)**

**rA**: 5'-PPP-rGrGrC rArArC rψrψrψ rGrArψ rCrCrC rψrCrG rGrψrψ rψrArG rCrGrC rCrGrG rCrCrψ rψrψrψ rCrψrC rCrCrA rCrArC rψrψrψ rCrArC rG -3'

**rB**: 5'-PPP-rGrGrG rArArA rψrψrψ rCrGrψ rGrGrψ rArGrG rψrψrψ rψrGrψ rψrGrC rCrCrG rψrGrψ rψrψrC rψrArC rGrArψ rψrArC rψrψrψ rGrGrψ rC -3'

**rC**: 5'-PPP-rGrGrA rCrArψ rψrψrψ rCrGrA rGrArC rArGrC rArψrψ rψrψrψ rψrCrC rCrGrA rCrCrψ rψrψrG rCrGrG rArψrψ rGrψrA rψrψrψ rψrArG rG -3'

**rD**: 5'-PPP-rGrGrC rGrCrψ rψrψrψ rGrArC rCrψrψ rCrψrG rCrψrψ rψrArψ rGrψrC rCrCrC rψrArψ rψrψrC rψrψrA rArψrG rArCrψ rψrψrψ rGrGrC rC -3'

**rE**: 5'-PPP-rGrGrG rArGrA rψrψrψ rArGrψ rCrArψ rψrArA rGrψrψ rψrψrA rCrArA rψrCrC rGrCrψ rψrψrG rψrArA rψrCrG rψrArG rψrψrψ rGrψrG rψ -3'

**rF**- 5'-PPP-rGrGrG rArψrC rψrψrψ rArCrC rψrArC rCrArC rGrψrψ rψrψrG rCrψrG rψrCrψ rCrGrψ rψrψrG rCrArG rArArG rGrψrC rψrψrψ rCrCrG rA -3'

**DNA cubes**

**dA**: 5'-OH-GGC AAC TTT GAT CCC TCG GTT TAG CGC CGG CCT TTT CTC CCA CAC TTT CAC G -3'

**dB**: 5'-OH-GGG AAA TTT CGT GGT AGG TTT TGT TGC CCG TGT TTC TAC GAT TAC TTT GGT C -3'

**dC**: 5'-OH-GGA CAT TTT CGA GAC AGC ATT TTT TCC CGA CCT TTG CGG ATT GTA TTT TAG G -3'

**dD**: 5'-OH-GGC GCT TTT GAC CTT CTG CTT TAT GTC CCC TAT TTC TTA ATG ACT TTT GGC C -3'

**dE**: 5'-OH-GGG AGA TTT AGT CAT TAA GTT TTA CAA TCC GCT TTG TAA TCG TAG TTT GTG T -3'

**dF**: 5'-OH-GGG ATC TTT ACC TAC CAC GTT TTG CTG TCT CGT TTG CAG AAG GTC TTT CCG A -3'

**DNA cubes with rUs in the corners**

**dA:** 5'-OH-GGC AAC rUrUrU GAT CCC TCG GrUrU rUAG CGC CGG CCrU rUrUT CTC CCA CAC rUrUrU CAC G -3'

**dB:** 5'-OH-GGG AAA rUrUrU CGT GGT AGG TrUrU rUGT TGC CCG TGrU rUrUC TAC GAT TAC rUrUrU GGT C -3'

**dC:** 5'-OH-GGA CAT rUrUrU CGA GAC AGC ArUrU rUTT TCC CGA CCrU rUrUG CGG ATT GTA rUrUrU TAG G -3'

**dD:** 5'-OH-GGC GCT rUrUrU GAC CTT CTG CrUrU rUAT GTC CCC TArU rUrUC TTA ATG ACT rUrUrU GGC C -3'

**dE:** 5'-OH-GGG AGA rUrUrU AGT CAT TAA GrUrU rUTA CAA TCC GCrU rUrUG TAA TCG TAG rUrUrU GTG T -3'

**dF:** 5'-OH-GGG ATC rUrUrU ACC TAC CAC GrUrU rUTG CTG TCT CGrU rUrUG CAG AAG GTC rUrUrU CCG A -3'

**DNA cubes with PS (all Ts)**

**dA**: 5'-OH-GGC AAC T*T*T* GAT* CCC T*CG GT*T* T*AG CGC CGG CCT* T*T*T* CT*C CCA CAC T*T*T* CAC G -3'

**dB**: 5'-OH-GGG AAA T*T*T* CGT* GGT* AGG T*T*T* T*GT* T*GC CCG T*GT* T*T*C T*AC GAT* T*AC T*T*T* GGT* C -3'

**dC**: 5'-OH-GGA CAT* T*T*T* CGA GAC AGC AT*T* T*T*T* T*CC CGA CCT* T*T*G CGG AT*T* GT*A T*T*T* T*AG G -3'

**dD**: 5'-OH-GGC GCT* T*T*T* GAC CT*T* CT*G CT*T* T*AT* GT*C CCC T*AT* T*T*C T*T*A AT*G ACT* T*T*T* GGC C -3'

**dE**: 5'-OH-GGG AGA T*T*T* AGT* CAT* T*AA GT*T* T*T*A CAA T*CC GCT* T*T*G T*AA T*CG T*AG T*T*T* GT*G T* -3'

**dF**: 5'-OH-GGG AT*C T*T*T* ACC T*AC CAC GT*T* T*T*G CT*G T*CT* CGT* T*T*G CAG AAG GT*C T*T*T* CCG A -3'

**DNA cubes with PS (all Cs and Ts)**

**dA**: 5'5'-OH-GGC* AAC* T*T*T* GAT* C*C*C* T*C*G GT*T* T*AG C*GC* C*GG C*C*T* T*T*T* C*T*C* C*C*A C*AC* T*T*T* C*AC* G -3'

**dB**: 5'-OH-GGG AAA T*T*T* C*GT* GGT* AGG T*T*T* T*GT* T*GC* C*C*G T*GT* T*T*C* T*AC* GAT* T*AC* T*T*T* GGT* C* -3'

**dC**: 5'-OH-GGA C*AT* T*T*T* C*GA GAC* AGC* AT*T* T*T*T* T*C*C* C*GA C*C*T* T*T*G C*GG AT*T* GT*A T*T*T* T*AG G -3'

**dD**: 5'-OH-GGC* GC*T* T*T*T* GAC* C*T*T* C*T*G C*T*T* T*AT* GT*C* C*C*C* T*AT* T*T*C* T*T*A AT*G AC*T* T*T*T* GGC* C* -3'

**dE**: 5'-OH-GGG AGA T*T*T* AGT* C*AT* T*AA GT*T* T*T*A C*AA T*C*C* GC*T* T*T*G T*AA T*C*G T*AG T*T*T* GT*G T* -3'

**dF**: 5'-OH-GGG AT*C* T*T*T* AC*C* T*AC* C*AC* GT*T* T*T*G C*T*G T*C*T* C*GT* T*T*G C*AG AAG GT*C* T*T*T* C*C*G A -3'

**SUPPORTING FIGURES**

**
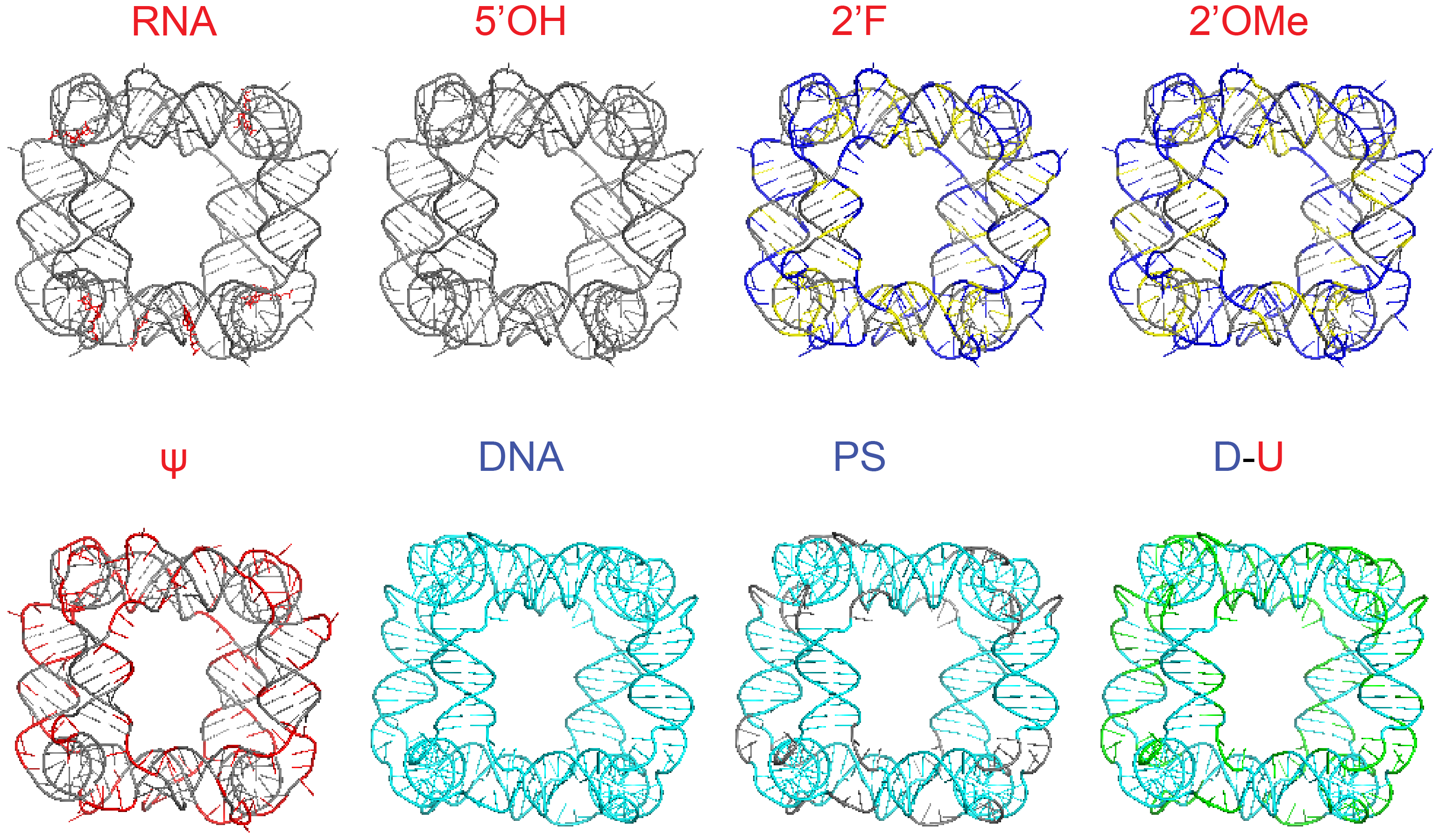
**

**Figure S1.** 3D models of chemically modified NANPs. For RNA cube, 5′-triphosphates are shown in red. For 2′F and 2′OMe modified cubes, fluorinated and methylated pyrimidines are depicted in blue and yellow, respectively. For ψ cubes, ψ (5-ribosyluracil) replacing Us are shown in red. For PS cubes, thymidine phosphorothioates are highlighted in black. For D-U cubes, green indicates sites where Ts were replaced with Us at single-stranded cube corner regions.


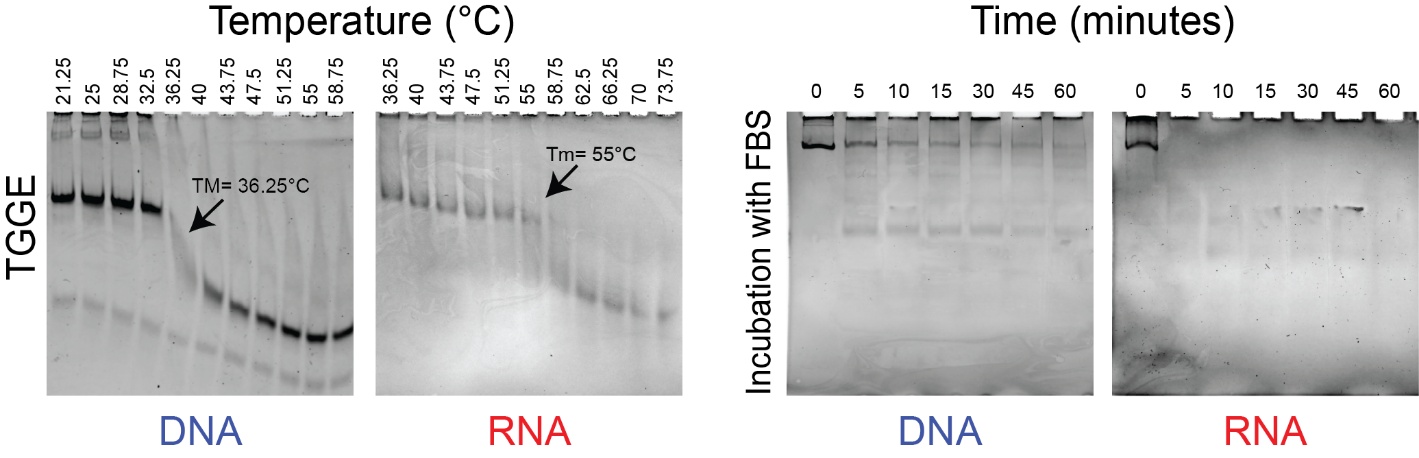


**Figure S2.** Representative gels assessing the thermal and enzymatic stability of NANPs. Thermal gradient gel electrophoresis (TGGE) shows the temperature-dependent stability of the cubes. Enzymatic stability was evaluated by incubating NANPs in 10% fetal bovine serum (FBS).


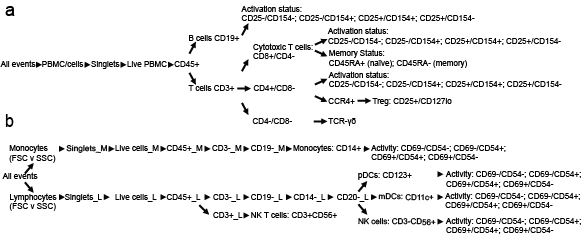


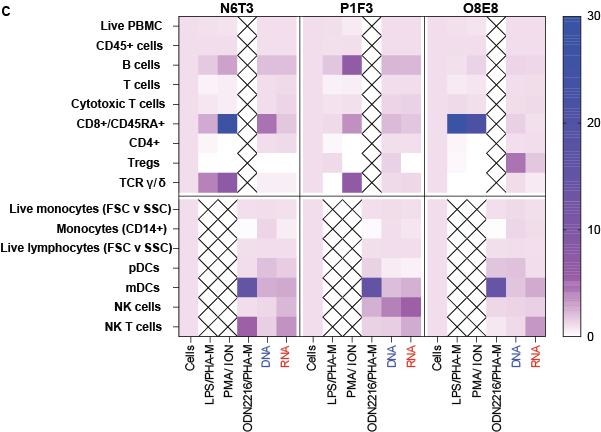


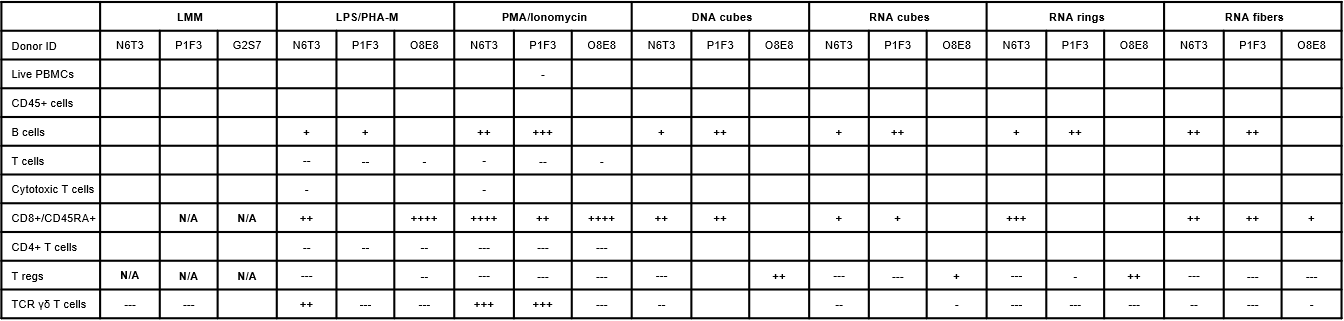

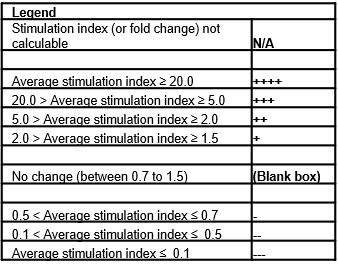
**d**

**e**

**
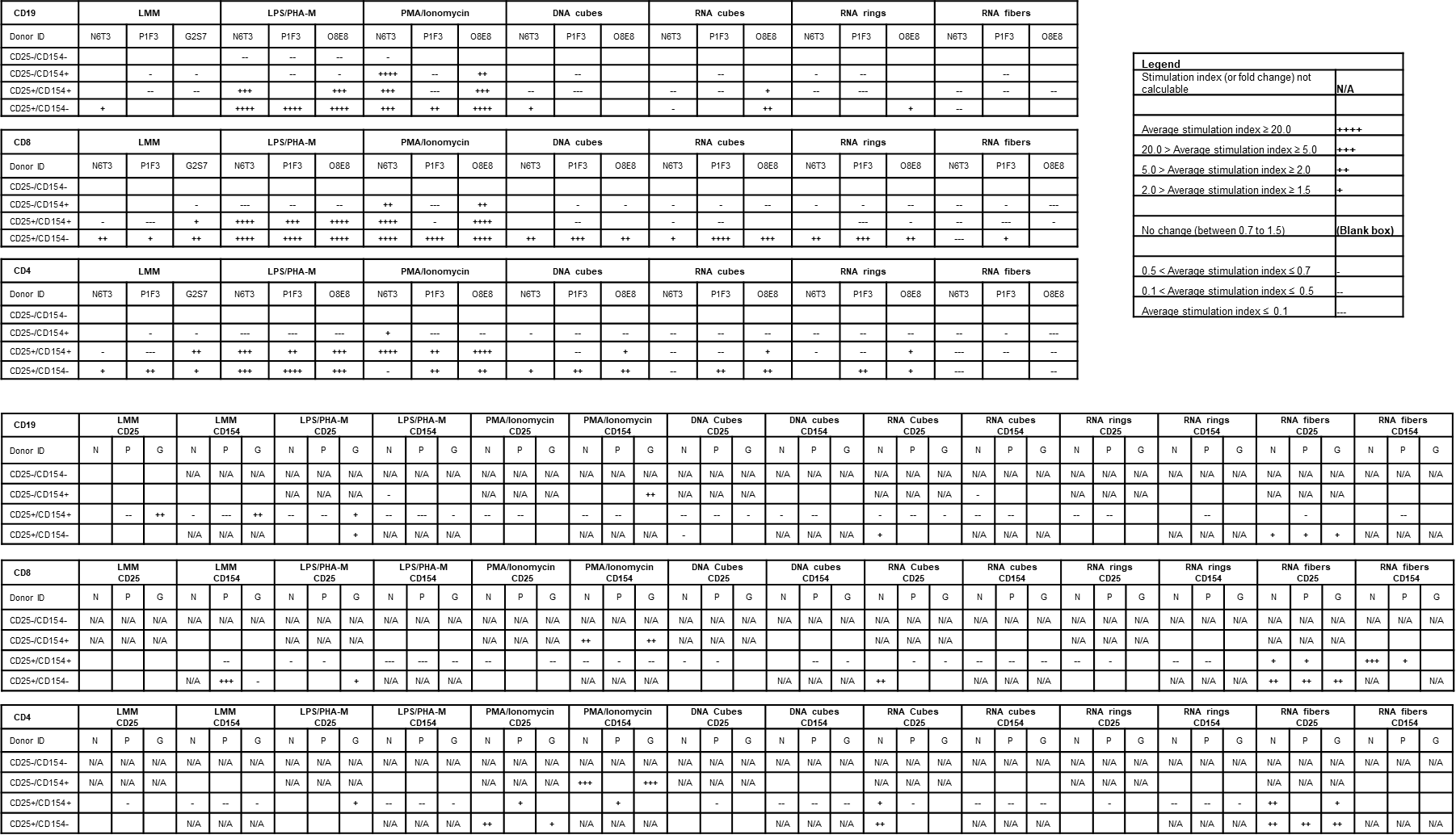
**

**f**

**
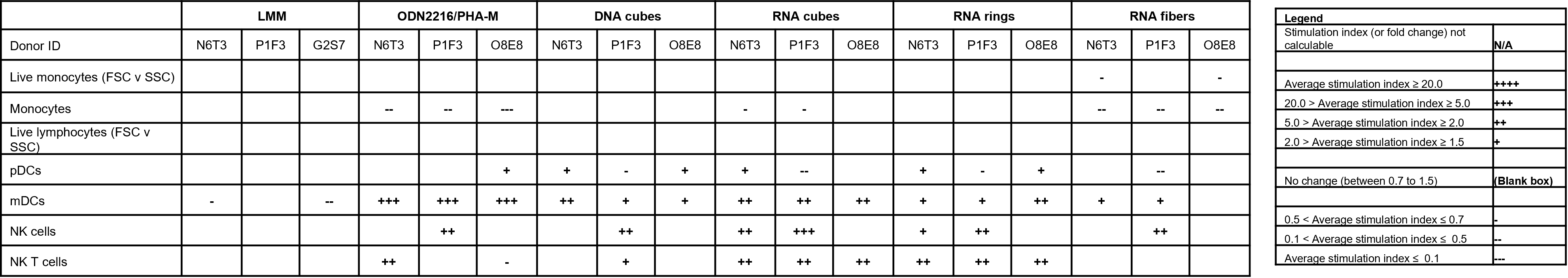
**

**g**

**
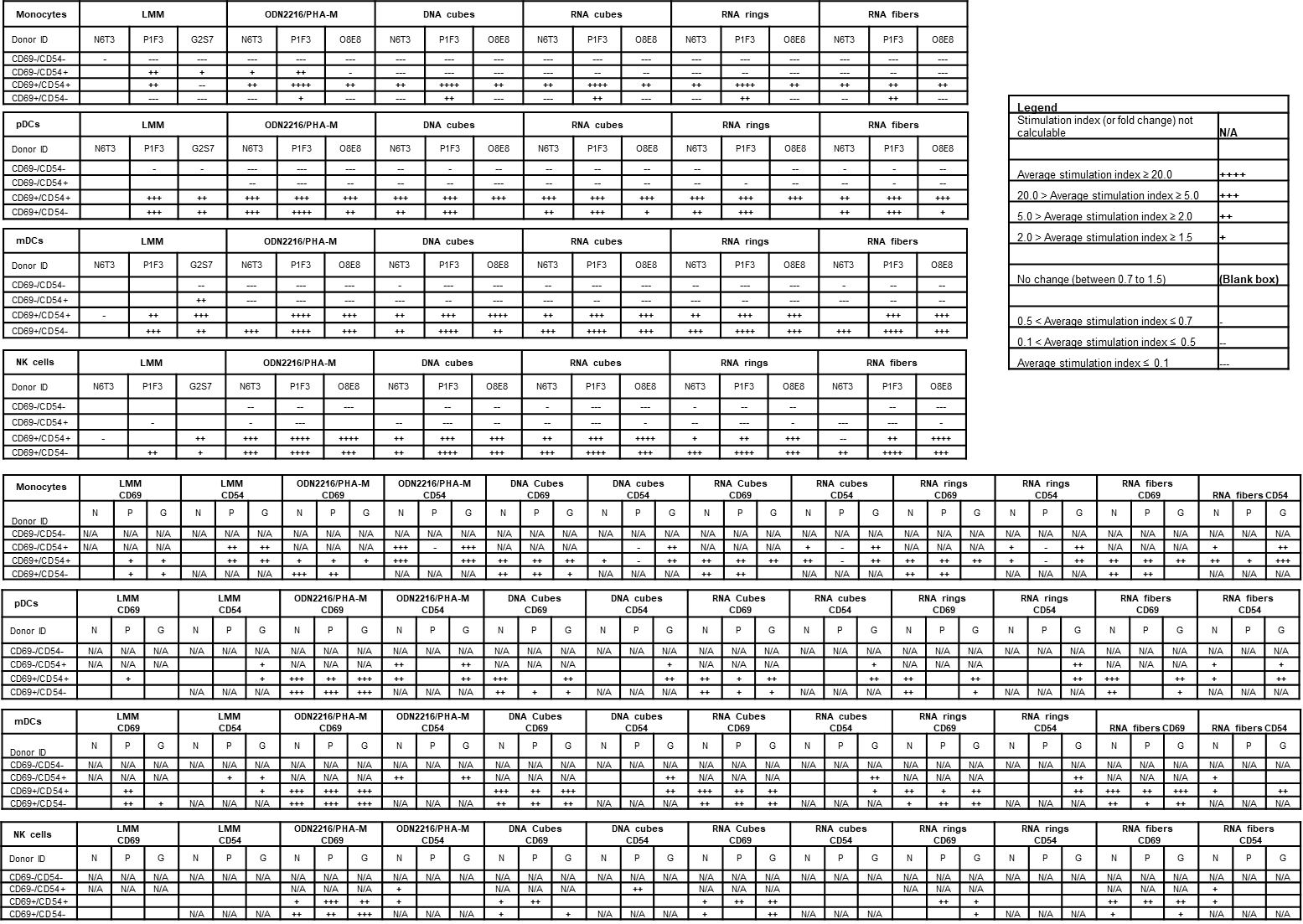
**

**Figure S3:** Immunophenotyping of NANPs. (**a**) The gating strategy for the immunophenotyping panel #1 shows subsequent gates created from each parent gate. All events are gated via forward and side scatter to obtain the main gate comprising PBMC or cells. The PBMC gate is then gated to include singlets. From the singlet population, all dead cells are excluded, and live cells remain. The live PBMC are then gated on CD45+ cells. B cells (or CD19+ cells) are then identified out of the CD45+ gate and activity defined by the presence/absence of CD25 and CD154 is determined for the CD19+ cells. T cells (or CD3+ cells) are also defined from the CD45+ gate. From the CD3+ gate, CD4 and CD8 markers are used to define CD8+/CD4- T cells (cytotoxic T cells), CD4+/CD8- T cells, and CD4-/CD8- T cells. The activity of both CD8+/CD4- T cells (cytotoxic T cells) and CD4+/CD8- T cells is then defined by the presence/absence of CD25 and CD154. The memory status of cytotoxic T cell cells is also defined using the presence of CD45RA (naïve). CD4+/CD8- T cells are also further gated by CD25 and CD127 to define regulatory T cells as CD25+/CD127low. CD4-/CD8- T cells are further gated on TCR-γδ to define the TCR-γδ T cells**.** (**b**) The gating strategy for the immunophenotyping panel #2 shows subsequent gates created from each parent gate. All events are gated via forward and side scatter to obtain two different populations identified as monocytes and lymphocytes. The monocyte population defined by FSC and SSC are gated further to include only singlets and then exclude dead cells. To identify the CD14+ cells (monocytes), the live cells are gated on CD45+ cells, CD3- cells, CD19- cells, and CD14+. The activity of CD14+ cells are determined by the presence and/or absence of CD69 and CD54. The lymphocyte population defined by FSC and SSC are gated further to include only singlets and then exclude dead cells. Live cells are then gated on CD45+. CD45+ population is then gated on CD3. The CD3- cell population is further gated on CD19-, CD14-, and CD20-. From this population (lymphocyte FSC vs SSC, singlets, live cells, CD45+, CD3-, CD19-, CD14-, CD20-), pDCs (CD123+), mDCs (cD11c+), and NK cells (CD56+) are defined. The activity of each of these cell populations is determined by the presence and/or absence of CD69 and CD54. The CD3+ cell population is used to define NK T cells (CD3+ CD56+). (**c**) Heat maps represent stimulation indexes of cell population percentages in untreated/negative controls, LMM control/vehicle control, positive controls: LPS/PHA-M; PMA/Ionomycin, ODN2216/PHA-M and NANP-treated samples (10 nM DNA cubes; 10 nM RNA cubes). The stimulation indexes were calculated compared to the untreated negative controls. Each box in the heat map represents the average of two values for each of the treatment/cell population combinations. Data from cultures obtained from each of the three donors are represented side-by-side. (**d**, **e**, **f**, **g**) Tables represent stimulation indices of cell population percentages in untreated/negative controls, LMM control/vehicle control, positive controls: LPS/PHA-M; PMA/Ionomycin, ODN2216/PHA-M and NANP-treated samples (10 nM DNA cubes; 10 nM RNA cubes; 10 nM RNA rings; 10 nM RNA fibers). The stimulation indices were calculated by comparing the treatment to the untreated negative control: (**d**) and (**e**) are for panel 1, and (**f**) and (**g**) are for panel 2. The plus and minus symbols in the legend tables represent the same data present in the corresponding heat maps (Figure 3) but show stratified groups of stimulation index values as indicated in the legend boxes. Due to the low abundance of some cell populations in the PBMC, these data should be treated semi-quantitatively/qualitatively. The sequences of RNA rings and RNA fibers are listed in PMCID: PMC6540121 (DOI: 10.1021/acs.nanolett.8b01283).


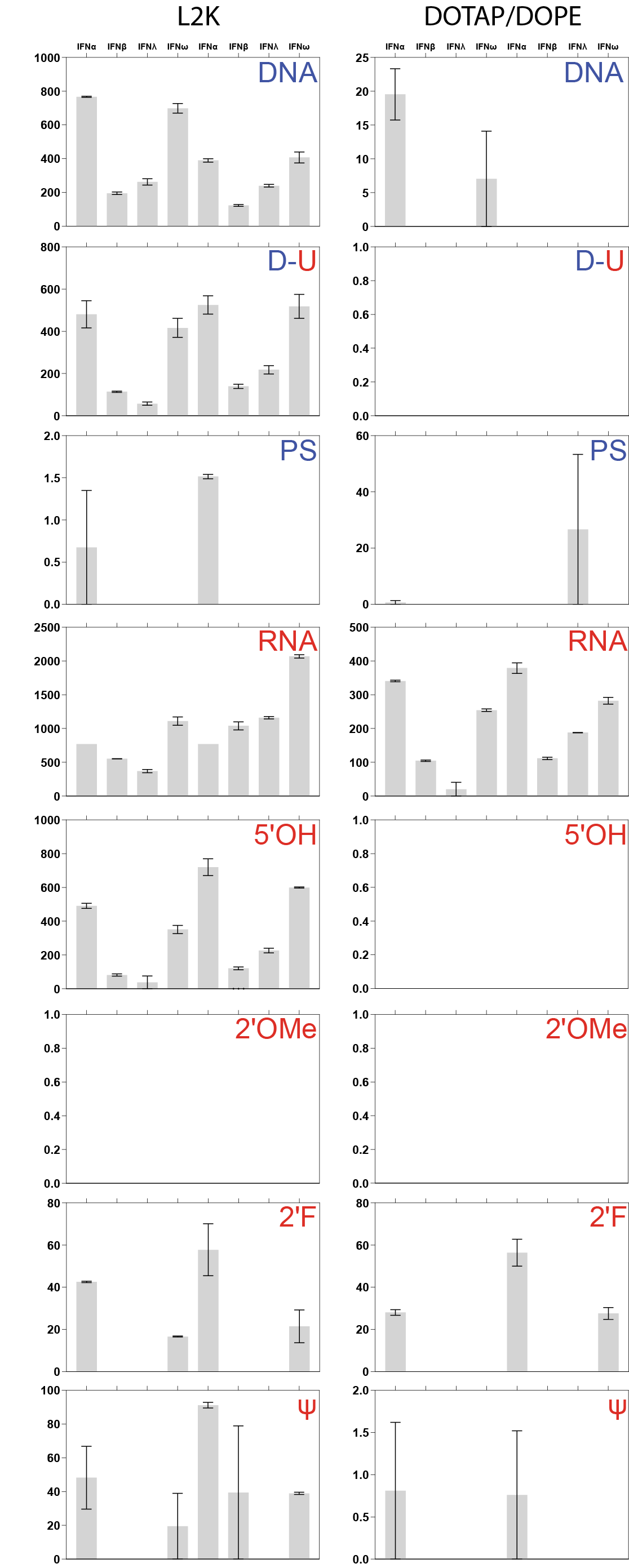


**Figure S4:** Induction of type I and III interferons following transfection of 5′PPP- or 5′OH-RNA NANPs using either Lipofectamine 2000 (L2K) or DOTAP/DOPE. Data represents the mean responses from two donors, error bars show the range. Nomenclature of cube variants: DNA – DNA cubes; PS – DNA cubes with backbone phosphorothioate modifications; D-U – DNA cubes containing ssUs at the corners; 5′OH – RNA cubes with a hydroxyl group at the 5′ end; RNA – RNA cubes with a 5′-triphosphate group. The remaining RNA cubes carried 5′-triphosphates and ribose modifications, where the 2′OH was substituted with either fluorine (2′F) or a 2′-O-methyl group (2′OMe), or uridine was substituted with pseudouridine (ψ).


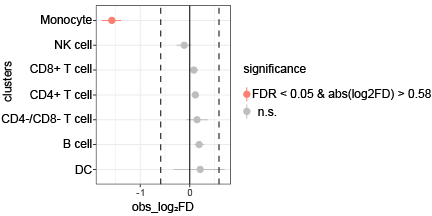


**Figure S5:** Single cell sequencing captured cells from PBMC after RNA cube exposure shows that only the monocyte population has a statistically significant decrease in the number of cells compared to negative control.

**
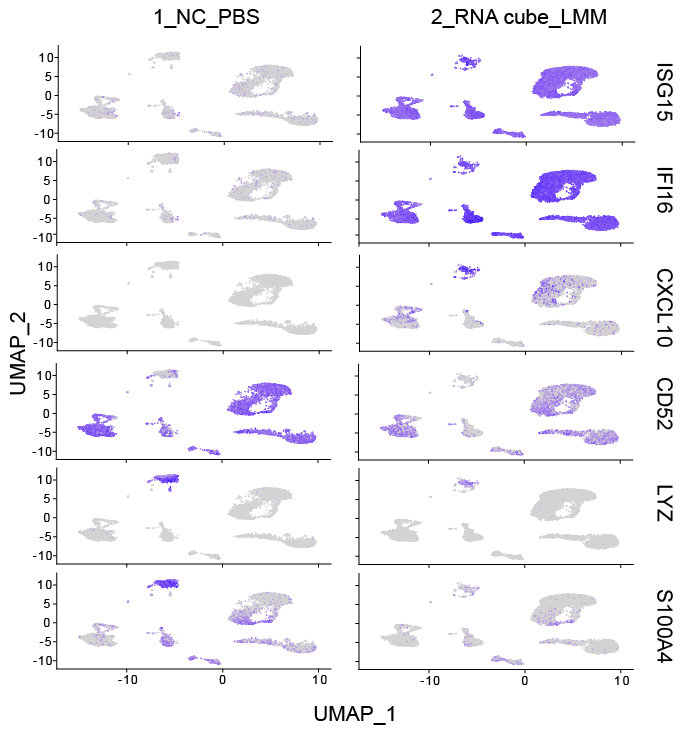
**

**Figure S6:** Examples of differentially expressed genes between PBMC with RNA cube exposure and negative control include ISG15, IFI6, CXCL10, CD52, LYZ, and S100A4

**
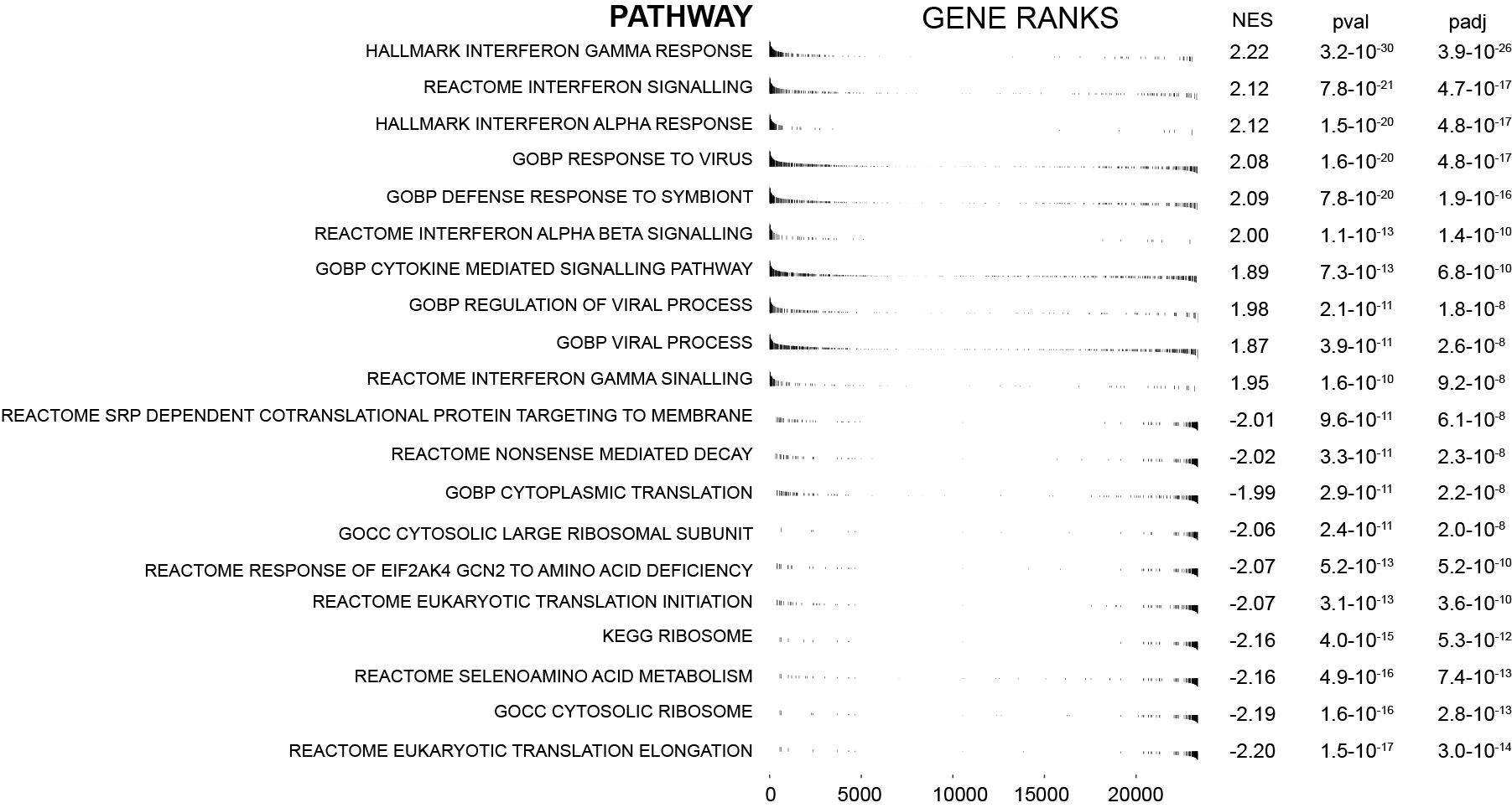
**

**Figure S7:** Gene Set Enrichment Analysis result shows the top twenty pathways statistically significantly affected by the RNA cube exposure included pathways involved in interferon signaling and cellular responses to a virus.


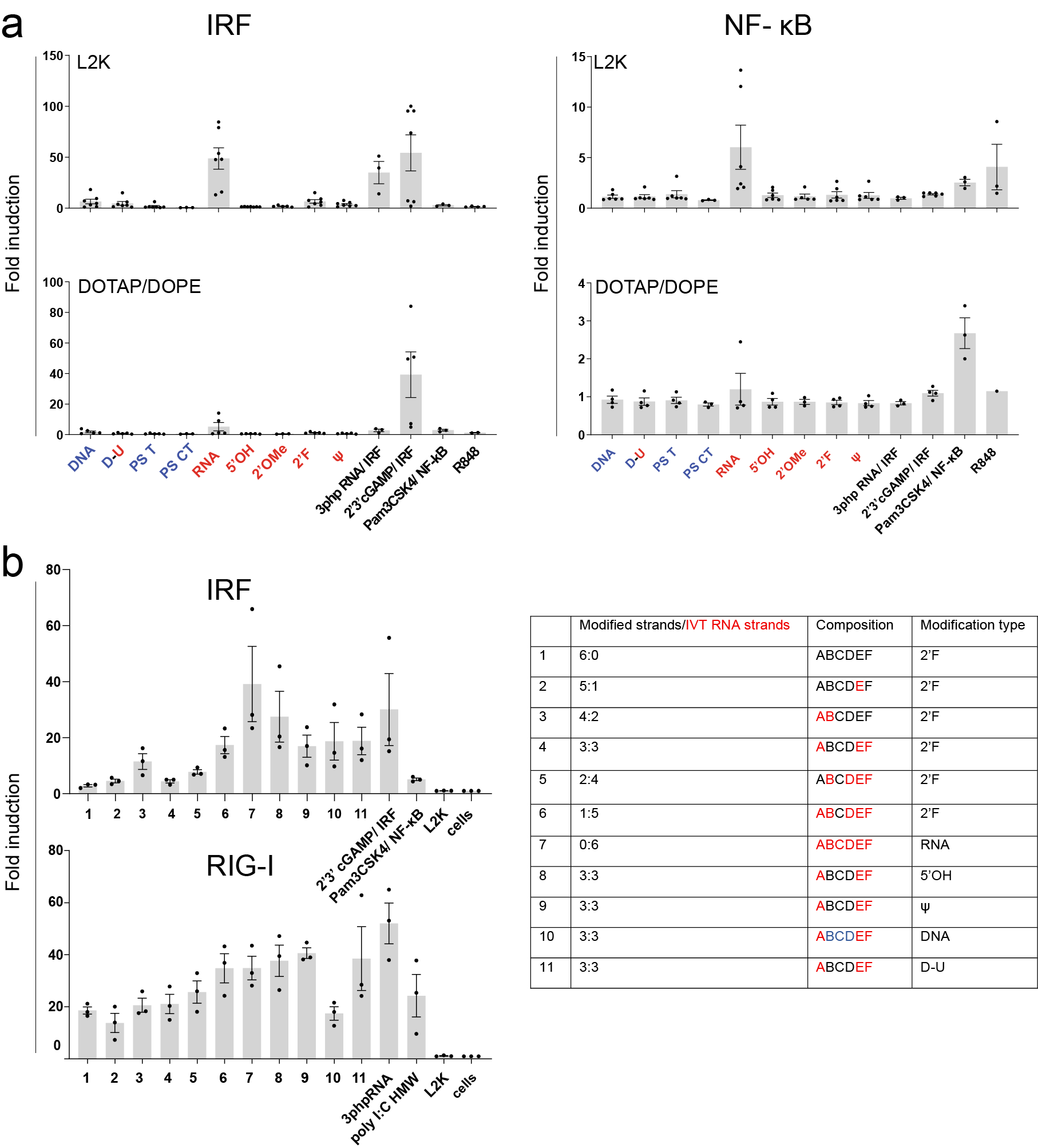


**Figure S8:** Activation of signaling pathways leading to induction of luciferase or SEAP under the control of IRF or NF-κB in THP1-Dual reporter cells based on the cube modification (**a**) and ratio of modified monomers to RNA monomers in THP1 Dual cells (IRF) and HEK-Lucia RIG-I (RIG-I) (**b**). Data are presented as mean ± SEM, N=7 for (**a**) and N=3 for (**b**). Nomenclature of cube variants: DNA – DNA cubes; PS – DNA cubes with backbone phosphorothioate modifications; D-U – DNA cubes containing ssUs at the corners; 5′OH – RNA cubes with a hydroxyl group at the 5′ end; RNA – RNA cubes with a 5′-triphosphate group. The remaining RNA cubes carried 5′-triphosphates and ribose modifications, where the 2′OH was substituted with either fluorine (2′F) or a 2′-O-methyl group (2′OMe), or uridine was substituted with pseudouridine (ψ). Each bar represents the mean response ± SEM; sample sizes were n = 3-7 for (**a**) and n = 3 for (**b**).


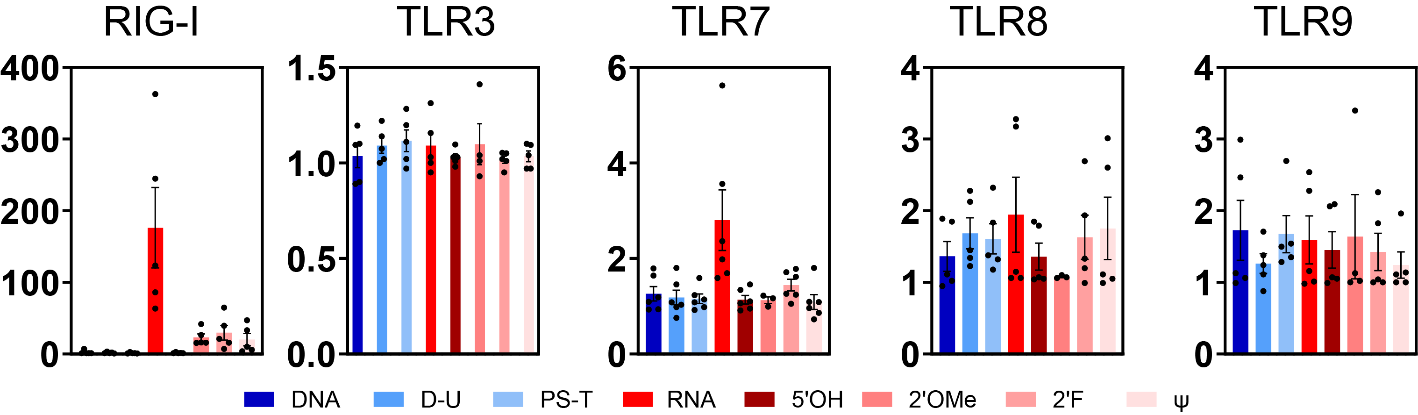


**Figure S9:** Production of luciferase following RIG-I stimulation in HEK-Lucia RIG-I cells, and SEAP production after stimulation of the corresponding TLRs in HEK-Blue cells. Data are presented as mean ± SEM (N = 5). Nomenclature of cube variants: DNA – DNA cubes; PS – DNA cubes with backbone phosphorothioate modifications; D-U – DNA cubes containing ssUs at the corners; 5′OH – RNA cubes with a hydroxyl group at the 5′ end; RNA – RNA cubes with a 5′-triphosphate group. The remaining RNA cubes carried 5′-triphosphates and ribose modifications, where the 2′OH was substituted with either fluorine (2′F) or a 2′-O-methyl group (2′OMe), or uridine was substituted with pseudouridine (ψ). Each bar shows the mean response ± SEM (n = 5-6).


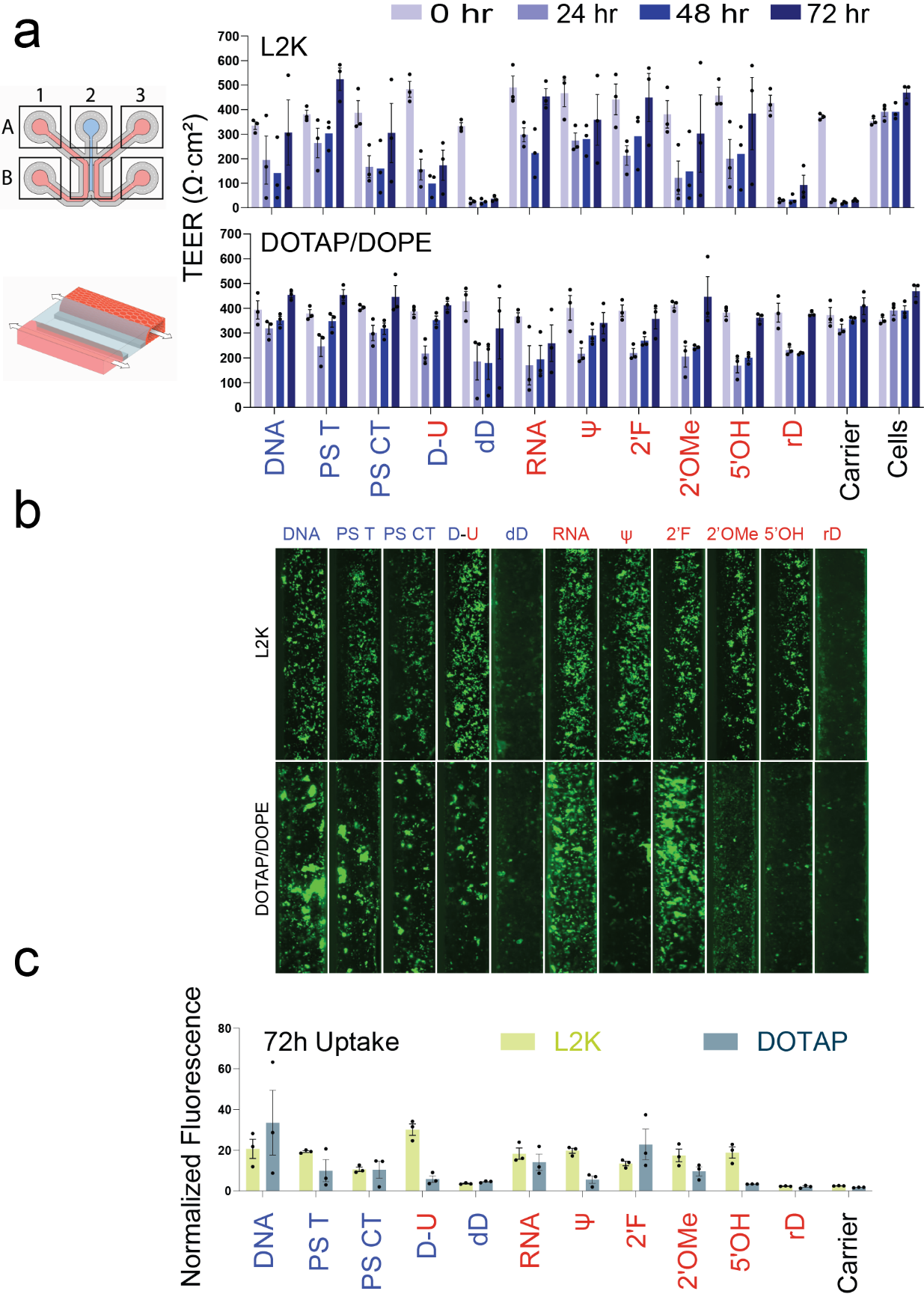


**Figure S10:** Layout of a 3L64 OrganoReady® Colon Caco-2 chip, showing the inlets (1A, 2A, 3A), the outlets (1B, 3B), and the observation window (2B), and demonstrating how the Caco-2 tubule is seeded against the collagen-I (a). Transepithelial electrical resistance (TEER) measurements following treatment with the cubes panel, complexed with L2K or DOTAP/DOPE. Control conditions include the carrier alone. TEER values are presented as mean ± SEM, (n=3) (b). Representative microscopy images of OrganoReady® Colon Caco-2 cultures at 4x magnification, showing fluorescent uptake of the cubes panel at 72 hr. (c) Normalized fluorescence for the 72h uptake of the cubes panel is graphed, the intensity of the Al488 signal was quantified and presented as mean ± SD, (n=3). Nomenclature of cube variants: DNA – DNA cubes; PS – DNA cubes with backbone phosphorothioate modifications; D-U – DNA cubes containing ssUs at the corners; 5′OH – RNA cubes with a hydroxyl group at the 5′ end; RNA – RNA cubes with a 5′-triphosphate group. The remaining RNA cubes carried 5′-triphosphates and ribose modifications, where the 2′OH was substituted with either fluorine (2′F) or a 2′-O-methyl group (2′OMe), or uridine was substituted with pseudouridine (ψ). dD and rD denote ssDNA and ssRNA cube monomers D, respectively. Each bar represents the mean response ± SEM (n = 3).

## SUPPORTING METHODS

## *Peripheral Blood Mononuclear Cells (PBMC) Isolation and Culture*

### *PBMC Isolation.* PBMC were isolated from healthy human donor whole blood collected in BD Biosciences li-heparin vacutainers (San Jose, CA, USA) under NCI-Frederick protocol OH9-C-N046. An expanded step by step protocol for the PBMC isolation is described in detail in NCL Protocol ITA-37.1 and ITA-37.2.^1^ In brief, the collected whole blood was diluted at equal volume with PBS and then layered over Ficoll-Paque Premium solution. Density gradient centrifugation was then used to separate out the mononuclear cell layer. This mononuclear cell layer was collected and washed twice with HBSS. The PBMC were resuspended in RPMI-1640 medium supplemented with 10% heat inactivated FBS, 100 U/mL penicillin, 100 µg/mL streptomycin, and 2 mM L-glutamine (complete RPMI-1640 medium), counted, and the cell concentration was adjusted to 1.25x10^6^ cells/mL.

### *PBMC activation and NANP treatment.* PBMC were cultured at a final concentration of 1x10^6^ cells/mL (final volume 1 mL) in a 24-well plate in a 37˚C/95% CO_2_ incubator for approximately 20 hours. Untreated (or unstimulated) PBMC were used as negative controls and consisted of 800 uL of PBMC (1.25x10^6^ cells/mL) and 200 uL of complete RPMI-1640 media. Positive controls consisted of PBMC activated with 20 ng/mL LPS + 10 µg/mL PHA-M, 50 ng/mL PMA + 1 µg/mL Ionomycin, or 5 µg/mL ODN2216 + 10 µg/mL PHA-M.

### Stock NANPs (DNA cubes, RNA cubes, RNA rings, and RNA fibers) were complexed with the lipofectamine carrier (LMM). An aliquot of 100 uL of 1 uM NANP stock was combined with 20 uL of LMM and mixed well by pipetting. Each NANP/LMM complex was then incubated for 5-30 minutes at room temperature followed by dilution with 1.88 mL OptiMEM medium (2 mL total; 50 nM NANP). The vehicle/carrier control (LMM control) was prepared in the same manner: OptiMEM medium was combined with LMM (at a ratio of 5-parts medium: 1-part LMM) and then incubated at room temperature for 5- 30 minutes and diluted further with OptiMEM medium to obtain LMM at the same concentration as in the complexed samples. The complexed NANPs or prepared LMM control (200 uL) were then added to the PBMC (800 uL PBMC at 1.25x10^6^ cells/mL) at a dilution of 5 (MRD5). The final concentration of the NANPs in each PBMC sample was 10 nM in 1 mL. LMM controls were run a separate day as the NANP and positive control samples due to limited sample quantities for preparation. However, when LMM controls were run they were processed alongside additional negative controls.

## *Multicolor Flow Cytometry.*

## *Antibody titration.* NCL protocol ITA-37.1 describes the titration procedure using both compensation beads and PBMC.^1^ Additionally, the titration performed for the two immunophenotyping panels here within is the same titration used for the method development of the panels described previously.^1^ In brief, a six-point calibration curve (stock, dilution (Dil) 5, 25, 125, 625, 3125) for each antibody or dye used in the panels was prepared. To perform the titrations for the antibodies and viability dye, 10-fold dilution of UltraComp eBeads Plus compensation beads in staining buffer and PBMC (50% live/50% dead) at approximately 5x10^5^ cells/mL in 1X PBS were used, respectively. An aliquot of 50 µL diluted beads or PBMC was added to 96-well plate wells (1 well per antibody or dye per calibration point, respectively). The antibody and dye dilutions were then prepared using staining buffer or 1X PBS, and 5 µL of antibody or dye dilution was added to the wells containing the 50 µL beads or PBMC (additional MRD of approximately 10), respectively. The plate was then incubated at room temperature for 30 minutes in the dark, centrifuged, followed by the supernatant removal. The samples were then resuspended in staining buffer and run on the NovoCyte 3005 (Agilent; Santa Clara, CA). The necessary parameters were selected as indicated in NCL protocol ITA-37.1.^1^ Data analysis was then performed in NovoExpress. An overlay histogram with all dilutions of a single antibody or dye was made with each fluorophore on the x-axis and count on the y-axis.

### *Single stain controls.* Single stain controls were prepared as indicated in NCL protocol ITA-37.1 and were the same single stain control samples run for some of the samples in the earlier study.^2^ In brief, the optimal dilutions as determined by the titrations described above were used to perform single stain controls. As with the titrations, the antibody single stains utilized 10-fold dilution of UltraComp eBeads Plus compensation beads in staining buffer and the viability dye single stain utilized PBMC (50% live/50% dead) at approximately 5x10^5^ cells/mL in 1X PBS. An aliquot of 50 µL of diluted beads or PBMC were added to 96-well plate wells (one well per antibody or dye, respectively). An aliquot of 5 µL of optimal antibody or dye dilution was added to corresponding well with 50 µL beads or PBMC, respectively. The samples were then incubated for 30 minutes, centrifuged, followed by the supernatant removal. Samples were then resuspended in staining buffer (or 1X PBS). The samples were then run on the NovoCyte 3005. The necessary parameters were selected as indicated in NCL protocol ITA-37.1 using the auto compensation setting in the NovoCyte software ^3^.

### *Fluorescence minus one (FMO) controls.* FMOs were prepared as indicated in NCL protocol ITA-37.1 and were the same FMOs run for some of the samples in the previous study.^2^ In brief, PBMC activated with PMA/ionomycin or ODN2216/PHA-M (as described above) were used for panel 1 and 2 FMOs, respectively. Samples needed to conduct the FMO controls for each panel were unstained, fully stained sample with labeling antibodies and each FMO control (all labeling antibodies or dye minus one). The PBMC samples were spiked with heat-shocked cells so that the viability dye could successfully stain a portion of each sample. The samples were washed with 1X PBS and then resuspended in 49.2 µL 1X PBS. The appropriate samples (fully stained sample and all FMOs except Zombie Aqua dye FMO) were stained with Zombie Aqua dye at a final concentration that correlated to the optimal dilution corrected for cell concentration and staining volume. After incubation the samples were washed with staining buffer and incubated with appropriately prepared antibody master mixes as indicated in NCL protocol ITA-37.1 to allow for the proper optimal concentration for each antibody with corrections for cell concentration and staining volume in each sample. The samples were then washed with staining buffer, fixed with 2% PFA, washed again, and resuspended in staining buffer for acquisition. The Novocyte 3005 was used to perform data acquisition. The parameters (channels and laser intensities) used for this acquisition were the same as those used in the single stain controls and are indicated in NCL protocol ITA-37.1. Data analysis and proper compensation was performed in NovoExpress and described in NCL protocol ITA-37.1.

### *Immunophenotyping panels.* The immunophenotyping protocol is detailed in NCL protocol ITA-37.2.^4, 5^ Untreated (negative control), activated (positive controls), and NANP-treated PBMC were used to run the two panels. The master mixes for each panel were prepared using the optimal antibody dilution corrected for cell concentration and staining volume for each individual antibody. Each panel had a labeling antibody master mix and an isotype control master mix (of equal concentration). The cell samples were washed twice with 1X PBS and all appropriate samples (samples to be stained with labeling antibody master mixes) were stained with Zombie Aqua dye for 30 minutes at room temperature. The samples were then washed twice with staining buffer and then incubated for 30 minutes with the appropriate amount of corresponding master mix/staining buffer (final staining volume: 100 µL). Finally, the samples were washed with staining buffer, fixed with 2% PFA for 15 minutes at room temperature, washed twice more with staining buffer, resuspended in 500 uL staining buffer, and stored at 4˚C until data acquisition.

### Data acquisition was performed on the NovoCyte 3005 with the same applied parameters as the corresponding FMO control experiments. The minimum volume of the sample collected was set to 300 µL, and the maximum number of events was collected given the stop volume. Compensation obtained from the FMO panels was applied post-acquisition and the files were analyzed in FCS Express 7. A detailed step-by-step description of the gating process is provided in the NCL protocol ITA-37.2. The negative gates in each treatment condition were set using the corresponding isotype control samples to account for any shifts in signal generated by the different treatment conditions. Immune cell subsets were analyzed for (1) population percentages in relation to a defined parent population and (2) geometric mean fluorescence intensities (gMFIs). For panel 1, the Live PBMC population was calculated as a percentage of “PBMC/cells” also referred to as the main gate. Other cell population percentages, such as B cells, T cells, etc. were calculated in relation to Live PBMC population not in relation to the immediate parent gate. When percentages of cells in each activation quadrant were however determined by comparison to the immediate parent gate. For example, CD25-/CD154- CD19+ cells were defined as a percentage of the CD19+ cells. For panel 2, the live monocyte population and live lymphocyte population were calculated as a percentage of “Monocytes (FSC v SSC)” and “Lymphocyte (FSC v SSC)” populations, respectively. Monocytes (CD14+) cell population was determined as a percentage of the live monocytes. The DC, NK and NK T cell populations were defined as a percentage of live lymphocytes. Percentages of cells in each activation quadrant were determined by comparison to the immediate parent gate. The population percentages and gMFIs for each treatment group were then compared the untreated (negative control) values to determine stimulation indexes (or fold changes). FCS Express 7, Microsoft Excel, and Graph Pad Prism were used for data analysis and visualization.

**Supporting References**

1. <https://www.cancer.gov/nano/research/ncl/protocols-capabilities>.

2. Newton, H. S.; Zhang, J.; Donohue, D.; Unnithan, R.; Cedrone, E.; Xu, J.; Vermilya, A.; Malys, T.; Clogston, J. D.; Dobrovolskaia, M. A., Multicolor flow cytometry-based immunophenotyping for preclinical characterization of nanotechnology-based formulations: an insight into structure activity relationship and nanoparticle biocompatibility profiles. *Front Allergy* **2023,** *4*, 1126012.

3. Newton, H. S.; Zhang, J.; Dobrovolskaia, M. A., NCL Method ITA-37.1: Immunophenotyping: Instrument Calibration and Reagent Qualification for Immunophenotyping Analysis of Human Peripheral Blood Mononuclear Cell Cultures. **2022**.

4. Newton, H. S.; Zhang, J.; Dobrovolskaia, M. A., Immunophenotyping: Instrument Calibration and Reagent Qualification for Immunophenotyping Analysis of Human Peripheral Blood Mononuclear Cell Cultures: Version 3. In *National Cancer Institute's Nanotechnology Characterization Laboratory Assay Cascade Protocols*, Bethesda (MD), 2005.

5. Newton, H. S.; Zhang, J.; Dobrovolskaia, M. A., Immunophenotyping: Analysis of Nanoparticle Effects on the Composition and Activation Status of Human Peripheral Blood Mononuclear Cells: Version 3. In *National Cancer Institute's Nanotechnology Characterization Laboratory Assay Cascade Protocols*, Bethesda (MD), 2005.
